# In or out of reach? Long-term trends in the reach of health assessments in the Swedish occupational setting

**DOI:** 10.5271/sjweh.4192

**Published:** 2024-12-01

**Authors:** Elin Ekblom-Bak, Magnus Lindwall, Linnea Eriksson, Andreas Stenling, Magnus Svartengren, Robert Lundmark, Lena Kallings, Erik Hemmingsson, Daniel Väisänen

**Affiliations:** 1 Department of Physical Activity and Health, The Swedish School of Sport and Health Sciences, Stockholm, Sweden.; 2 Department of Psychology, University of Gothenburg, Gothenburg, Sweden.; 3 Department of Psychology, Umeå University, Umeå, Sweden.; 4 Department of Sport Science and Physical Education, University of Agder, Kristiansand, Norway.; 5 Department of Medical Sciences, Occupational and Environmental Medicine, Uppsala University, Uppsala, Sweden.; 6 Department of Occupational and Environmental Medicine, Uppsala University Hospital, Uppsala, Sweden.; 7 Department of Health, Education and Technology, Luleå University of Technology, Luleå, Sweden.; 8 Department of Public Health and Caring Sciences, General practice, Uppsala University, Uppsala, Sweden.

**Keywords:** health, occupational health service, representativeness, Sweden, workplace

## Abstract

**Objectives:**

This study aimed to investigate the reach of a large-scale health assessment delivered by the occupational health service in Sweden for almost 30 years.

**Methods:**

A total of 418 286 individuals who participated in a health assessment (Health Profile Assessment, HPA) between 1995–2021 were included. A comparative sample was obtained from Statistics Sweden, comprising the entire working population for each year (4 962 127 to 6 011 829 unique individuals per time period). Sociodemographic and work organization characteristics were compared between the HPA and comparative population for six different periods. Under- and overrepresented groups in the private and public sectors were identified using the most recent data (2015–2021).

**Results:**

With negative per cent indicating underrepresentation, the most notable changes over time in representation in the HPA population compared to the comparative were observed for women (-1.2% to -12.8%), private sector employees (-9.4% to 14.9%), individuals with ≥3 years of employment (14.5% to 0.9%), in personal care (0.8% to -8.8%) and manufacturing (0.7% to 6.4%) occupations. Consistently overrepresented groups (median representation across periods) included individuals who had a single income source (6.3%) and were middle-aged (10.8%), born in Sweden (5.9%), associate professionals (8.7%), and employed in companies with high operating profit (17.9%) and low staff turnover (14.3%). Conversely, individuals with low income (-34.0%) and employed in small companies/organizations (-10.9%) were consistently underrepresented. Middle-aged women in education occupations were most underrepresented in the public sector, while in the private sector, it was young women in service and shop sales occupations.

**Conclusions:**

This health assessment has reached many professionals, including hard-to-reach groups, but did not fully represents the Swedish workforce throughout the years.

Health inequality has a major impact on society via systematic differences between socioeconomic groups in morbidity and mortality (1), as well as workplace productivity loss and sick leave (2), and may be mitigated by targeting behavioral factors, including physical activity and diet (2, 3). Therefore, strengthening the prevention of ill health and promoting healthy behaviors has been targeted as priority areas to reduce social inequalities in health (4).

The workplace has the potential to be an important setting for health promotion and disease prevention as it may reach diverse sociodemographic groups otherwise unengaged in health programs or health assessments outside of the occupational setting. Health-promoting activities aim to improve the health of the workers and/or target organizational aspects (5) and are either initiated by the organization themselves or in close collaboration and with support from the occupational health service. In Sweden, occupational health services operate in a free market (6). Three major players dominate the market, holding approximately half of the market share. The occupational health services offer various health assessments, which mainly target lifestyle behaviors and health at an individual level (7). The first health assessment with a standardized method and procedure was developed during the 1970s and 1980s (8, 9) to evaluate lifestyle behaviors and health parameters and support the employee in maintaining or improving health. This method, the Health Profile Assessment (HPA), has since then been delivered by the occupational health service to employees at companies and organizations all around Sweden. From the mid-1990s, almost 800 000 HPA have been registered in a central database.

While the effectiveness of health-promoting workplace programs has been implied (10), there is a general underlying challenge in the reach of these activities (11). Previous research has reported low-to-moderate participation rates, often <60% (11–14), with men, low-wage workers, and individuals with precarious work being less likely to participate (7, 12, 15, 16). As these underrepresented groups also have been concluded to have an increased risk of poor health and a lower prevalence of healthy behaviors (17–19), as well as shorter healthy working life expectancy (20), this is a cause for concern. Most previous studies describing reach have compared participation within one or a few targeted workplaces, and only a few studies have investigated participation in a representative sample of the general population (16, 21, 22). Also, potential differences between the public and private sectors in availability and health-related policies have been suggested (23, 24). To further understand the reach and representativeness of health-promoting initiatives in the occupational setting, there is a need for additional studies investigating the reach over time while comparing it to a large and representative sample of the working population. The standardized delivery of the HPA to Swedish employees enables investigation of this, which may provide important knowledge into addressing health inequalities within an occupational context.

This study aims to investigate the reach of a large-scale health assessment delivered by the occupational health service in Sweden for almost 30 years. More specifically, the primary aim is to compare the sociodemographic and work-organizational characteristics of individuals participating in a HPA over time, with individuals of the general working population during the same time periods. The secondary aim is to identify under- and overrepresented groups, based on sex, age, and occupation, in the private and public sectors, respectively, using the most recent data from the years 2015 to 2021.

## Methods

This study used a descriptive repeated cross-sectional design. Data were obtained from the HPA database, containing information from HPA conducted by occupational health services. To understand the selection of individuals in the database, it is relevant to understand the typical decision-making process that precedes offering employees a health assessment in Sweden. First, the employer decides to provide a health assessment for their staff. Following this, the employer contracts a health assessment provider, typically an occupational health service. If the provider includes the HPA in its service portfolio, which ≈90% of Sweden’s occupational health services do, the employer and provider may then agree to conduct the HPA. This option is chosen for about 25% of all health assessments performed by occupational health services in Sweden. Finally, employees are informed about the offer and given the choice of whether to participate. Participation in an HPA is voluntary and free of charge for the employee. After participating in the HPA, all data are registered in a central database. This allows occupational health service personnel to compare results with reference materials, track progress over time, and generate aggregated reports for all participants at a specific workplace. Also, participants can monitor their results and compare them with reference material. The database is managed by the HPI Health Profile Institute, a private company, which has also been responsible for the development and standardization of the HPA method since the beginning. The standardization of the HPA involves a well-documented procedure, comprehensive 3–4 days training and examination of the coaches (mainly occupational health service nurses or physiotherapists), and the use of software that guides the process and supports the coaches. The HPA method comprises essentially three parts: (i) self-reported data (questionnaire on physical activity, lifestyle habits, perceived health, and symptoms); (ii) physical examination and tests measuring body mass, height, waist circumference, blood pressure, and cardiorespiratory fitness; and (iii) behavior change dialogue (the HPA concludes with a 20–40 minute behavior change dialogue with the HPA coach using a motivational interviewing approach). The Stockholm Ethics Review Board approved the study (Dnr 2015/1864-31/2; 2016 9-32; 2019-05711; 2023-07086-02), which follows the principles of the Declaration of Helsinki. All participants provided informed consent before HPA data collection. The ethical board classified the research as register-based, with no requirement for additional consent from the participants.

### Participants

Employers offer employees a single HPA or additional follow-up HPA, leading to multiple tests per individual in the database, for some individuals. However, for this study, we included only one test per individual. From 1 January 1990 to 31 December 2021, the database contained 436 918 unique individual HPA. No verification of completion of the HPA’s three main components was available in the database. To ensure completion of the HPA, inclusion criteria required data on at least two of six core questionnaire variables (exercise, smoking, diet, perceived health, perceived stress, and perceived back/neck pain) and two of five physical examination variables (estimated VO_2_max, height, weight, systolic and diastolic blood pressure). A total of 12 327 records were excluded for not meeting these criteria. Moreover, the annual rate of HPA in the early years was considerably lower compared to the following, assessments before 1995 (N=1508) were excluded to address discrepancies in early data. Individuals <18 years (N=168) and those without verified salary (N=4588) or source of salary (N=3789) were excluded. In total, 418 286 individuals aged 18–85 years (N=1237 aged ≥66 years) were included (see supplementary material, www.sjweh.fi/article/4192, figure S1 for exclusion details). Unfortunately, there is no record of how many workers have been offered participation but declined.

To study reach we used representativeness, defined as the comparison of a sample to the target audience, as the operational construct (25). A comparative sample from the Swedish working population was retrieved from Statistics Sweden (26). For each year from 1995 to 2021, it comprised all men and women aged ≥18 who had been employed sometime during this period, which resulted in a total of 243 440 352 data rows, where an individual contributed with one data row for each year as employed. Out of these, 127 403 387 had a verified salary from work performed and employment status. Analyses were performed in six time periods (1995–1999, 2000–2004, 2005–2009, 2010–2014, 2015–2019, 2020–2021) with individuals included only once per period they were in employment (randomly selected which employment year was included), resulting in 4 962 127 to 6 011 829 unique individuals per time period. This comparative dataset was used for the primary aim analyses. For the secondary aim, a 10% stratified sample from the full comparative sample was drawn for each year (2015–2021), ensuring each individual appeared only once. This corresponds to approximately 50% of the total comparative population in any given year and was selected to ensure representativeness across key demographic groups, allowing for a comparison of populations.

### Procedures

Anthropometrics and lifestyle variables were collected during the HPA. Sociodemographic and work organization characteristics were derived from Statistics Sweden using linkage with participants’ personal identification numbers. Height and weight were measured in light-weighing clothing and body mass index was calculated. Cardiorespiratory fitness was assessed as estimated VO_2_max via the Åstrand submaximal cycle ergometer tests (27). Perceived physical work situation, exercise habits, smoking, and perceived health were self-reported and categorized (see supplementary table S1 for full statement, original, and aggregated categories). Any episode of sickness absence due to any cause (>14 days in the previous year from the HPA) was retrieved from The Swedish Social Insurance Agency.

Place of birth was categorized as Sweden, Europe, and outside of Europe. Civil status was categorized into 'partner' versus 'single'. Municipality of residence was categorized into three groups: metropolitan, dense, and rural municipalities, according to the Swedish Agency for Economic and Regional Growth (28). Educational level was obtained as years of education and grouped into tertiary (≥13 years), secondary (10–12 years), and primary education (≤9 years). Occupation data (available from 2001) were categorized according to the Swedish Standard Classification of Occupation, which is based on the International Standard Classification of Occupation (29). Occupation was defined with both major and sub-major groups (see supplementary table S2). Due to heterogeneity within the ten major occupational groups, sub-major groups were identified a priori to the analyses based on variation in contact with clients/patients/students or occupational physical activity level. We have in previous studies used a similar categorization (17, 30). Annual income from employment was categorized into five categories based on percentages of the median in the population (31). Number of income sources was arbitrarily categorized into three groups (1, 2–3, >3). Contractual temporariness was dichotomized into having the same employer for <3 or ≥3 years (31).

Ownership sectors were divided into private, public governmental, and public regional (both region and municipality). Number of employees in the work organization was categorized into four groups (32). Economic sector of the work organizations was categorized according to the Swedish Standard Industrial Classification (33). Operating profit of each work organization was categorized into four groups based on percentages of the median in the population. The operating profit margin (the operating profit divided by the net sales) and annual staff turnover rate were both arbitrarily divided into four categories. The variables for operating profit and staff turnover rate were available from 1997 onwards (mainly private sector work organizations).

### Statistical analysis

**Figure 1:**
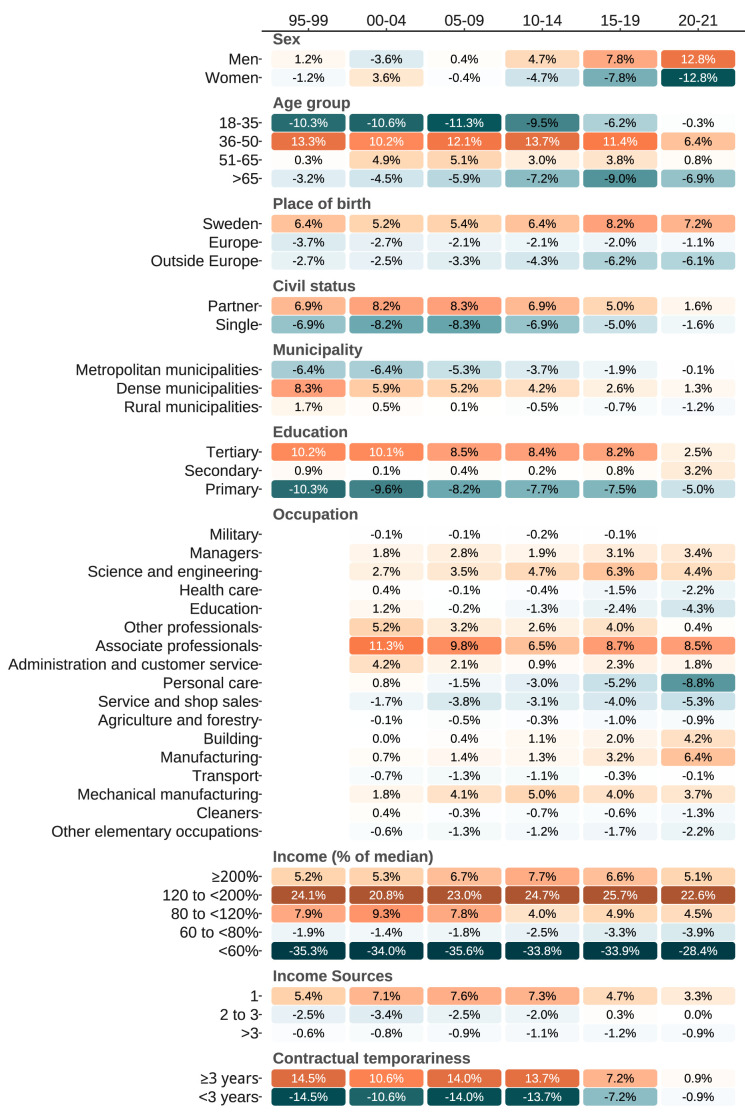
Sociodemographic characteristics over the study period among HPA participants compared to the comparative population. Positive differences indicate over-representation among HPA participants. Color gradients visually represent the magnitude of differences between the two populations. Specifically, white represents no difference, blue gradient represents negative values, and orange gradient represents positive values. As the difference between the databases increases, the shade of the color darkens, indicating a higher positive or negative difference.

**Figure 2:**
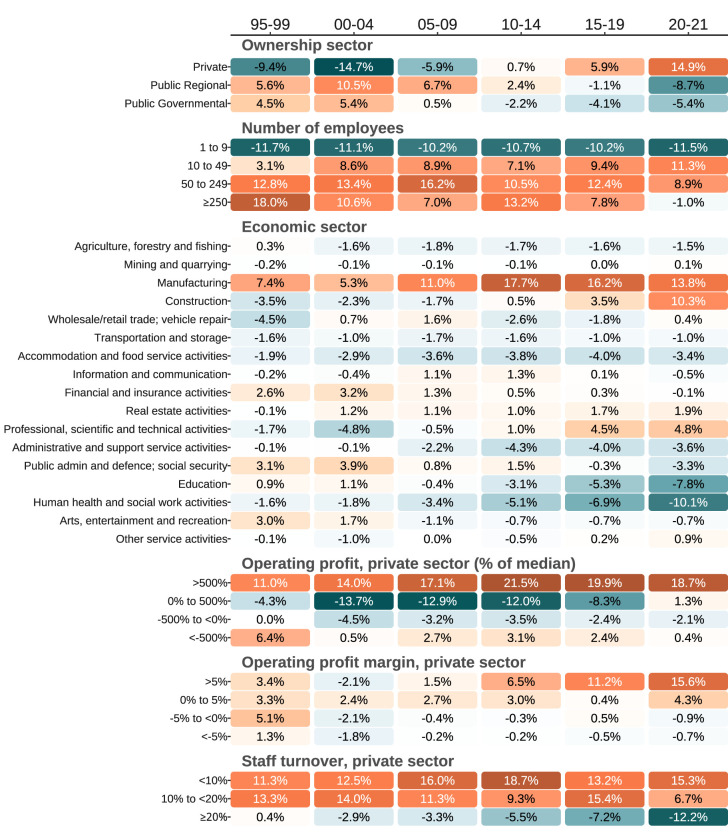
Work organization characteristics over the study period among HPA participants compared to the comparative population. Positive differences indicate over-representation among HPA participants. Color gradients visually represent the magnitude of differences between the two populations. Specifically, white represents no difference, blue gradient represents negative values, and orange gradient represents positive values. As the difference between the databases increases, the shade of the color darkens, indicating a higher positive or negative.

For the primary aim, sociodemographic and work organization characteristics of the HPA population are presented in tables 1 and 2, and absolute differences are further presented in figures 1 and 2. These analyses were based on all included data from the HPA population and the comparative population sample. Relative differences were calculated as (HPI data–comparative data)/comparative×100. For the secondary aim, combined groups based on sex, age groups (18–35, 36–50, 51–65 years), and occupational groups (occupational groups in table 1) were created within the HPA population using data from 2015 to 2021. This analysis was conducted separately for individuals in the public and private sectors, according to the following procedure: (i) data was grouped by all combinations of sex, age, and occupation; (ii) prevalence of these groups in the HPA population was compared with the comparative population; and (iii) k-means clustering (34) was used to identify underrepresented, represented, or overrepresented clusters in the HPA population in relation to the comparative population. The k-means clustering method iteratively assigns data points to clusters based on the squared Euclidean distance to centroids until cluster assignments stabilize. Hence, this method allows us to identify which combined groups (based on sex, age, and occupation) that were most different from each other in the HPA versus the comparative sample in terms of percentage point difference. The most under- and overrepresented combined groups are presented in table 3. All data curation was performed with R (35) using the Tidyverse package (36). The code can be found at https://github.com/dvaiman/WT_paper1.

**Table 1:** Sociodemographic characteristics over the study period among Health Profile Assessment participants (N=418 286). [**⸱⸱**=missing values].

Variables		1995–1999 (N=13 272)		2000–2004 (N=63 446)		2005-2009 (N=22 295)		2010-2014 (N=114 468)		2015-2019 (N= 85 950)		2020-2021 (N=18 855)
		N (%)		N (%)		N (%)		N (%)		N (%)		N (%)
Sex												
	Men	7005 (53)		30 435 (48)		63 576 (52)		64 428 (56)		51 387 (60)		12 134 (64)
	Women	6267 (47)		33 011 (52)		58 719 (48)		50 040 (44)		34 563 (40)		6721 (36)
	Missing	0 (0.0)		0 (0.0)		0 (0.0)		0 (0.0)		0 (0.0)		0 (0.0)
Age group (years)												
	18–35	4084 (31)		17 962 (28)		33 073 (27)		33 714 (29)		28 151 (33)		7048 (37)
	36–50	5989 (45)		25 735 (41)		50 999 (42)		49 117 (43)		34 054 (40)		6 837 (36)
	51–65	3197 (24)		19 704 (31)		37 994 (31)		31 186 (27)		23 348 (27)		4857 (26)
	>65	2 (0.0)		45 (0.1)		229 (0.2)		451 (0.4)		397 (0.5)		113 (0.6)
	Missing	0 (0.0)		0 (0.0)		0 (0.0)		0 (0.0)		0 (0.0)		0 (0.0)
Place of birth												
	Sweden	12 598 (95)		58 673 (92)		111 397 (91)		102 682 (90)		75 010 (87)		16 025 (85)
	Europe	536 (4.0)		3278 (5.2)		7331 (6.0)		7462 (6.5)		6290 (7.3)		1558 (8.3)
	Outside of Europe	138 (1.0)		1492 (2.4)		3564 (2.9)		4319 (3.8)		4645 (5.4)		1272 (6.7)
	Missing	0 (0.0)		3 (0.0)		3 (0.0)		5 (0.0)		5 (0.0)		0 (0)
Civil status												
	Partner	7005 (53)		32 599 (51)		61 080 (50)		54 178 (47)		38 711 (45)		7791 (41)
	Single	6267 (47)		30 847 (49)		61 215 (50)		60 290 (53)		47 239 (55)		11 064 (59)
	Missing	0 (0.0)		0 (0.0)		0 (0.0)		0 (0.0)		0 (0.0)		0 (0.0)
Municipality												
	Metropolitan	3083 (23)		15 157 (24)		31 248 (26)		32 637 (29)		27 132 (32)		6406 (34)
	Dense	6781 (51)		32 303 (51)		61 272 (50)		55 708 (49)		40 111 (47)		8541 (45)
	Rural	3353 (25)		15 877 (25)		29 703 (24)		26 123 (23)		18 707 (22)		3908 (21)
	Missing	55 (0.4)		109 (0.2)		72 (0.1)		0 (0.0)		0 (0.0)		0 (0.0)
Education												
	Tertiary	4777 (36)		25 355 (40)		50 807 (42)		51 210 (45)		40 825 (47)		8633 (46)
	Secondary	6452 (49)		30 544 (48)		58 276 (48)		53 373 (47)		39 130 (46)		8945 (47)
	Primary	2022 (15)		7458 (12)		13 022 (11)		9559 (8.4)		5616 (6.5)		1086 (5.8)
	Missing	21 (0.2)		89 (0.1)		190 (0.2)		326 (0.3)		379 (0.4)		191 (1.0)
Occupation												
	Military	**⸱⸱**		54 (0.1)		131 (0.1)		72 (0.1)		12 (0.0)		0 (0.0)
	Managers	**⸱⸱**		2975 (4.7)		8796 (7.2)		6395 (5.6)		6412 (7.5)		1654 (8.8)
	Science and engineering	**⸱⸱**		2929 (4.6)		7802 (6.4)		8230 (7.2)		7268 (8.5)		1315 (7.0)
	Healthcare	**⸱⸱**		1010 (1.6)		1968 (1.6)		1376 (1.2)		1620 (1.9)		360 (1.9)
	Education	**⸱⸱**		2844 (4.5)		5034 (4.1)		2688 (2.3)		2667 (3.1)		385 (2.0)
	Other professionals	**⸱⸱**		5413 (8.5)		9928 (8.1)		8073 (7.1)		9303 (11)		1805 (9.6)
	Associate professionals	**⸱⸱**		13 572 (21)		29 012 (24)		19 770 (17)		15 570 (18)		3697 (20)
	Administration and customer service	**⸱⸱**		6234 (9.8)		11 698 (9.6)		7170 (6.3)		7241 (8.4)		1690 (9.0)
	Personal care	**⸱⸱**		5595 (8.8)		11 899 (9.7)		6578 (5.7)		4572 (5.3)		659 (3.5)
	Service and shop sales	**⸱⸱**		1687 (2.7)		3676 (3.0)		3184 (2.8)		3414 (4.0)		777 (4.1)
	Agriculture and forestry	**⸱⸱**		256 (0.4)		830 (0.7)		689 (0.6)		591 (0.7)		179 (0.9)
	Building	**⸱⸱**		1756 (2.8)		5522 (4.5)		5162 (4.5)		4218 (4.9)		1475 (7.8)
	Manufacturing	**⸱⸱**		1996 (3.1)		5439 (4.4)		4332 (3.8)		6082 (7.1)		2053 (11)
	Transport	**⸱⸱**		755 (1.2)		1961 (1.6)		1554 (1.4)		1949 (2.3)		585 (3.1)
	Mechanical manufacturing	**⸱⸱**		3595 (5.7)		11 198 (9.2)		9444 (8.3)		5676 (6.6)		1260 (6.7)
	Cleaners	**⸱⸱**		1068 (1.7)		1755 (1.4)		890 (0.8)		958 (1.1)		132 (0.7)
	Other elementary occupations	**⸱⸱**		1467 (2.3)		3516 (2.9)		2275 (2.0)		1369 (1.6)		257 (1.4)
	Missing	13 272 (100)		10 240 (16)		2130 (1.7)		26 586 (23)		7028 (8.2)		572 (3.0)
Income (% of median)												
	≥200	1478 (11)		7333 (12)		15 786 (13)		15 978 (14)		10 599 (12)		2092 (11)
	120 –<200	6536 (49)		28 756 (45)		57 829 (47)		56 141 (49)		43 354 (50)		9223 (49)
	80–<120	3977 (30)		20 015 (32)		36 545 (30)		30 365 (27)		24 478 (28)		5885 (31)
	60–<80	743 (5.6)		3729 (5.9)		6466 (5.3)		5501 (4.8)		3989 (4.6)		879 (4.7)
	<60	538 (4.1)		3613 (5.7)		5669 (4.6)		6483 (5.7)		3530 (4.1)		776 (4.1)
	Missing	0 (0.0)		0 (0.0)		0 (0.0)		0 (0.0)		0 (0.0)		0 (0.0)
Number of income sources												
	1	10 363 (78)		49 053 (77)		94 013 (77)		87 699 (77)		62 640 (73)		14 007 (74)
	2–3	2671 (20)		13 346 (21)		26 358 (22)		25 182 (22)		22 012 (26)		4 605 (24)
	>3	238 (1.8)		1047 (1.7)		1924 (1.6)		1587 (1.4)		1298 (1.5)		243 (1.3)
	Missing	0 (0.0)		0 (0.0)		0 (0.0)		0 (0.0)		0 (0.0)		0 (0.0)
Contractual temporariness (years)												
	≥3	7779 (59)		34162 (54)		72203 (59)		66124 (58)		42398 (49)		8780 (47)
	<3	4937 (37)		27 189 (43)		45 716 (37)		43 200 (38)		41 041 (48)		9816 (52)
	Missing	556 (4.2)		2095 (3.3)		4376 (3.6)		5144 (4.5)		2511 (2.9)		259 (1.4)

**Table 2:** Work organization characteristics over the study period among Health Profile Assessment participants (N=418 286).

Variables		1995–1999 (N=13 272)		2000–2004 (N=63 446)		2005–2009 (N=122 295)		2010–2014 (N=114 468)		2015–2019 (N=85 950)		2020–2021 (N=18 855)
		N (%)		N (%)		N (%)		N (%)		N (%)		N (%)
Ownership sector												
	Private	6678 (50)		31 157 (49)		73 555 (60)		78 707 (69)		63 436 (74)		15 509 (82)
	Public governmental	1953 (15)		9084 (14)		10 186 (8.3)		5961 (5.2)		2611 (3.0)		365 (1.9)
	Public regional	4637 (35)		23 197 (37)		38 509 (31)		29 767 (26)		19 886 (23)		2979 (16)
	Missing	4 (0.0)		8 (0.0)		45 (0.0)		33 (0.0)		17 (0.0)		2 (0.0)
Number of employees												
	1–9	829 (6.2)		4295 (6.8)		9761 (8.0)		9758 (8.5)		7433 (8.6)		1903 (10)
	10–49	3216 (24)		19 311 (30)		37 841 (31)		33 842 (30)		27 828 (32)		7184 (38)
	50–249	4323 (33)		21 805 (34)		45 086 (37)		35 136 (31)		29 138 (34)		6407 (34)
	≥250	4348 (33)		15 940 (25)		25 231 (21)		30 588 (27)		19 040 (22)		3102 (16)
	Missing	556 (4.2)		2095 (3.3)		4376 (3.6)		5144 (4.5)		2511 (2.9)		259 (1.4)
Economic sector												
	Agriculture, forestry, & fishing	337 (2.5)		423 (0.7)		840 (0.7)		1231 (1.1)		855 (1.0)		147 (0.8)
	Mining and quarrying	1 (0.0)		67 (0.1)		124 (0.1)		141 (0.1)		151 (0.2)		60 (0.3)
	Manufacturing	3082 (23)		12 685 (20)		29 021 (24)		32 520 (28)		21 947 (26)		4351 (23)
	Construction	218 (1.6)		1828 (2.9)		5043 (4.1)		7681 (6.7)		8639 (10)		3272 (17)
	Wholesale & retail trade; repair of motor vehicles & motorcycles	980 (7.4)		8039 (13)		16 704 (14)		10 598 (9.3)		8397 (9.8)		2260 (12)
	Transportation and storage	365 (2.8)		2148 (3.4)		3762 (3.1)		3691 (3.2)		3143 (3.7)		693 (3.7)
	Accommodation & food service	206 (1.6)		440 (0.7)		542 (0.4)		847 (0.7)		471 (0.5)		86 (0.5)
	Information & communication	625 (4.7)		2885 (4.5)		6142 (5.0)		5451 (4.8)		3364 (3.9)		701 (3.7)
	Finance & insurance	601 (4.5)		3189 (5.0)		3735 (3.1)		2538 (2.2)		1678 (2.0)		324 (1.7)
	Real estate	275 (2.1)		2087 (3.3)		4049 (3.3)		3684 (3.2)		3495 (4.1)		775 (4.1)
	Professional, scientific & technical	736 (5.5)		2352 (3.7)		7674 (6.3)		7634 (6.7)		8897 (10)		2029 (11)
	Administrative & support service	16 (0.1)		73 (0.1)		1712 (1.4)		2334 (2.0)		2134 (2.5)		490 (2.6)
	Public administration & defense; compulsory social security	1100 (8.3)		5643 (8.9)		7028 (5.7)		7400 (6.5)		4202 (4.9)		487 (2.6)
	Education	1069 (8.1)		6377 (10)		11 586 (9.5)		8096 (7.1)		4518 (5.3)		573 (3.0)
	Human health & social work	2185 (16)		9234 (15)		14 716 (12)		11 817 (10)		7663 (8.9)		1159 (6.1)
	Arts, entertainment & recreation	793 (6.0)		3071 (4.8)		1982 (1.6)		1723 (1.5)		1401 (1.6)		274 (1.5)
	Other service activities	276 (2.1)		740 (1.2)		3250 (2.7)		2966 (2.6)		2732 (3.2)		672 (3.6)
	Missing	407 (3.1)		2165 (3.4)		4385 (3.6)		4116 (3.6)		2263 (2.6)		502 (2.7)
Operating profit (% of median)												
	>500	2925 (22)		21 002 (33)		45 278 (37)		48 116 (42)		34 008 (40)		7224 (38)
	0–500	1188 (9.0)		5353 (8.4)		15 792 (13)		16 861 (15)		16 926 (20)		5409 (29)
	-500–<0	479 (3.6)		1306 (2.1)		4939 (4.0)		4853 (4.2)		4774 (5.6)		1030 (5.5)
	<-500	1346 (10)		5159 (8.1)		9592 (7.8)		9715 (8.5)		5904 (6.9)		1093 (5.8)
	Missing	7334 (55)		30 626 (48)		46 694 (38)		34 923 (31)		24 338 (28)		4099 (22)
Operating profit margin (%)												
	>5	2149 (16)		12 525 (20)		33 121 (27)		37 948 (33)		32 880 (38)		8581 (46)
	0–5	1964 (15)		13 837 (22)		27 949 (23%)		27 029 (24)		18 054 (21)		4052 (21)
	-5–<0	1276 (9.6)		3564 (5.6)		7689 (6.3)		8221 (7.2)		6128 (7.1)		896 (4.8)
	<-5	549 (4.1)		2894 (4.6)		6842 (5.6)		6347 (5.5)		4550 (5.3)		1227 (6.5)
	Missing	7334 (55)		30 626 (48)		46 694 (38)		34 923 (31)		24 338 (28)		4099 (22)
Annual staff turnover rate (%)												
	<10	5521 (42)		26 023 (41)		55 555 (45)		54 900 (48)		31 886 (37)		8492 (45)
	10–<20	4792 (3)		23 586 (37)		42 969 (35)		38 510 (34)		34 813 (41)		7020 (37)
	≥20	2208 (17)		10 496 (17)		17 690 (14)		14 532 (13)		15 458 (18)		2790 (15)
	Missing	751 (5.7)		3341 (5.3)		6081 (5.0)		6526 (5.7)		3793 (4.4)		553 (2.9)

**Table 3:** Description of the nine most under- and overrepresented combined groups in the under- and overrepresented cluster by public and private sector. and overrepresented clustered groups in public and private sector. [HPA=Health Profile Assessment; M=men; W=women.]

Description of the combined groups		Prevalence in HPA population		Prevalence in comparative population		Prevalence difference
		%		%		%
**Underrepresented - Public**						
	W: 36–50 and Education	3.18		5.55		-2.37
	W: 51–65 and Personal care	5.56		7.57		-2.01
	W: 51–65 and Education	2.55		4.46		-1.91
	W: 18–35 and Personal care	4.82		6.59		-1.77
	W: 18–35 and Education	1.61		3.24		-1.63
	W: 36–50 and Health care	2.03		3.27		-1.24
	M: 18–35 and Personal care	0.82		2.01		-1.19
	W: 51–65 and Health care	1.56		2.72		-1.16
	M: 36–50 and Education	0.89		1.77		-0.88
**Underrepresented - Private**						
	W: 18–35 and Service & shop sales	0.66		4.86		-4.2
	M: 18–35 and Service & shop sales	0.75		3.39		-2.64
	W: 18–35 and Personal care	0.24		1.97		-1.73
	W: 36–50 and Service & shop sales	0.42		1.86		-1.44
	M: 18–35 and Other elementary occupations	0.49		1.57		-1.08
	W: 36–50 and Personal care	0.2		1.27		-1.07
	W: 51–65 and Service & shop sales	0.31		1.37		-1.06
	W: 18–35 and Other elementary occupations	0.16		1.22		-1.06
	W: 51–65 and Personal care	0.14		1.09		-0.95
**Overrepresented - Public**						
	W: 36–50 and Other professionals	4.58		3.22		1.36
	W: 36–50 and Administration & customer service	2.87		1.54		1.33
	M: 51–65 and Service & shop sales	1.78		0.85		0.93
	M: 36–50 and Associate professionals	2.57		1.64		0.93
	M: 36–50 and Service & shop sales	1.52		0.73		0.79
	W: 36–50 and Managers	2.16		1.39		0.77
	M: 51–65 and Associate professionals	2.23		1.46		0.77
	W: 51–65 and Administration & customer service	2.82		2.08		0.74
	M: 36–50 and Managers	1.62		0.91		0.71
**Overrepresented - Private**						
	M: 36–50 and Associate professionals	5.9		3.59		2.31
	M: 36–50 and Science & engineering	3.06		1.12		1.94
	M: 18–35 and Manufacturing	4.25		2.69		1.56
	M: 51–65 and Associate professionals	4.3		2.75		1.55
	M: 18–35 and Science & engineering	2.2		0.8		1.4
	M: 18–35 and Associate professionals	4.31		2.93		1.38
	M: 36–50 and Mechanical manufacturing	2.36		1.15		1.21
	W: 36–50 and Associate professionals	3.23		2.09		1.14
	W: 18–35 and Science and engineering	1.57		0.46		1.11

## Results

The number of HPA participants varied over the study period (table 1). Anthropometrics and lifestyle characteristics of the HPA population are presented in supplementary table S3. Regarding sociodemographic characteristics, the proportion of male participants increased, while the mean age remained stable over the study period (table 1). By the end of the study period compared to the beginning, there were lower proportions of HPA participants born in Sweden (85% versus 95%), in a relationship (41% versus 53%), and with temporary employment contracts (47% versus 59%). Conversely, the proportion of participants living in metropolitan municipalities (23% versus 34%) and with tertiary education (36% versus 46%) increased. Associate professionals (eg, technicians, insurance advisers, and finance and accounting occupations) remained the dominant occupational group throughout the study period and so did also the number of income sources.

Compared to the Swedish workforce, there was a constant overrepresentation in the HPA population of middle-aged individuals (median over periods, positive values indicate overrepresentation, 10.8%), those born in Sweden (5.9%), with a single income source (6.3%), associate professionals (8.7%), and with higher-than-median income (23.6%), with a constant underrepresentation for those with low income (-34.0%) (figure 1). Initially, women were only slightly underrepresented (-1.2%), but clearly underrepresented by the end (-12.8%). Changes over time included residents in dense municipalities (-6.4% to -0.1%), with tertiary education (10.2% to 2.5%), in those with ≥3 years of employment contract (14.5% to 0.9%), in personal care (0.8% to –8.8%) and manufacturing (0.7% to 6.4%) occupations. The underlying data for the comparative sample are presented in supplementary table S4. Relative differences are presented in supplementary table S5.

Regarding work organization characteristics, the proportion of work organizations in the private sector increased from 50% to 82%, with a shift from predominantly larger (≥50 employees) to smaller (10–49 employees) companies over the study period (table 2). The highest operating profit margins category also increased (16% to 46%). Participation in the economic sector Construction increased, and together with Manufacturing, these became the largest sectors by the end of the study period. The proportion of organizations with high relative operating profit and operating profit margin increased, which may partly be due to the increase in private sector participation and reduced missing data. Staff turnover rates remained stable over the study period.

Compared to the Swedish workforce, there was a shift in the HPA population from an underrepresentation of private sector employees at the beginning of the study period (-9.4%), to an overrepresentation by the end (14.9%) (figure 2). Micro-work organizations were constantly underrepresented (median over time periods, -10.9%). Individuals from work organizations with high operating profit (17.9%) and with low staff turnover (14.3%) were constantly overrepresented. By the end of the study period, the manufacturing and construction economic sectors were overrepresented in the HPA population (13.6%), while education and human health and social work activities were underrepresented (-7.8% and -10.1%). The underlying data for the comparative sample are presented in supplementary table S6. Relative differences are presented in supplementary table S7.

Table 3 presents the nine most underrepresented and overrepresented combined groups in the private sector and public sectors in the most recent years. Nine groups were presented as it was the least number of combined groups in one of the K-cluster analyses (for underrepresentation in the private sector). Within the public sector, the most underrepresented combined group was middle-aged women from education occupations. Other underrepresented combined groups were predominately women in either education or personal care occupations. Within the private sector, the most underrepresented combined group was young women from service and shop sales occupations, with other underrepresented groups also consisting of women in either service and shop sales; personal care; or other elementary occupations. All under-, over-, and represented combined groups are presented in supplementary tables S8 and 9, and further characteristics for these groups in supplementary tables S10–13 and figure S2.

## Discussion

The main findings in this study on the reach of a large-scale health assessment (the HPA) in the Swedish occupational setting were that the HPA has reached many working professionals across various sociodemographics and work organizations, including previously defined hard-to-reach groups. However, as should be expected, the HPA participants have not fully represented the Swedish workforce throughout the years, with variations between variables. For example, middle-aged individuals, born in Sweden, with single income source, associate professionals, and employees in companies with high operating profit and low staff turnover have been constantly overrepresented in the HPA population, while individuals with low income and in small companies/organizations have been consistently underrepresented. Notable changes in reach across the years were seen among women, private sector employees, with ≥3 years of employment, and employees in personal care and manufacturing occupations. Looking into combined groups, middle-aged women in education occupations were most underrepresented in the public sector, while in the private sector, it was young women in service and shop sales occupations.

We found that men were overrepresented in the HPA population, especially in recent years, contrasting with previous studies showing that women more often participate in workplace health promotion programs (7, 12). One explanation may be the overrepresentation of male-dominated economic sectors like manufacturing and construction, and the underrepresentation of female-dominated sectors including human health and social work activities sector in the most recent time periods. This is further supported by the combined group analyses, which showed that women were most underrepresented in both sectors, particularly in female-dominated occupations such as education and personal care (public sector), and service and shop sales and personal care (private sector). Additionally, this may be linked to the notable transition from an overrepresentation of public organizations in the earlier periods to private companies/organizations in more recent years. In Sweden, 'in-house' occupational health services in public organizations held a strong position in preventive and promotive work for employees during the 1990s and early 2000s. However, increased privatization of occupational health services, coupled with a more challenging economic environment for public sector organizations, has led to a shift in strategic focus over the past few decades. This shift has resulted in a greater emphasis on rehabilitation (reactive measures) rather than preventive initiatives in the public sector (personal communication with occupational health services). Previous research has shown mixed results regarding differences between public and private sectors, ranging from findings of no significant difference in the availability of workplace health promotion activities between private and public sectors (23) to more prevalent health-related policies available in the public sector (24). Moreover, we noted an increase in overrepresentation among employees from building and manufacturing occupations. This may be aligned with the current collective agreements in the Swedish construction industry, which include provisions for health examinations and working conditions (apart from the legislated and mandatory medical examination for risk-occupation employees) although the details can vary between agreements and arrangements.

We also noted a strong underrepresentation among young individuals in the earlier and middle time periods, which seems similar to other studies (7, 16, 37), with no underrepresentation in recent years. Possible explanations include an increased focus on and awareness of health and healthy living in today’s society, as well as a high prevalence of poor mental health in younger individuals (38). Also, an increase in lifestyle-related risk factors such as obesity and low cardiorespiratory fitness in recent years (39) may prompt individuals to seek more lifestyle-support initiatives, such as participating in occupational health examinations.

It was also notable that employees with low income, short contractual temporariness, and those born outside Europe were underrepresented over time. This is consistent with previous findings reporting that workers with nonstandard work arrangements (such as temporary, independent, or other contracts) are less likely to have access to workplace health promotion programs (16). Moreover, individuals from micro-organizations were underrepresented in the HPA population, which aligns with previous studies indicating that smaller work organizations have less availability of health-promoting activities (16, 23) and a lower percentage of affiliation to occupational health services (40). However, work organization size has also been negatively associated with participation in workplace health promotion programs (41). Higher operating profit was associated with overrepresentation in the HPA population. Although few studies have directly compared operating profit, previous research has shown that private companies with a better economic situation report more health-promoting activities (23). This may also help explain the underrepresentation of employees with short-term contracts and those born outside Europe, who are more often employed in low-margin businesses with limited resources for providing health examinations.

In addition to the sociodemographic and work organizational characteristics described in this study, other factors may influence participation in health promotion programs. Previous research indicates higher participation in programs that include multiple components (such as health assessments and additional health-promoting activities), target several behaviors, and offer incentives for participation (12). Additionally, the degree of leadership support (41) and the perception that participation is expected by colleagues and supervisors influence higher participation (42). It should also be noted that the employer has the responsibility to prevent work-related ill health, according to Swedish law (43). There is a risk that with too large a focus on individually based health assessments, the work environment is neglected, which may result in a decreased responsibility for work environment issues for the employer.

Strengths of the study include the utilization of data from standardized health assessments in the occupational setting conducted over almost three decades, and the comparison with a sample representing the Swedish working population for the same periods. Although Swedish national registries, including Statistics Sweden, are known for their high coverage and completeness (44), relying on the Swedish personal number to obtain sociodemographic and work organization data may have excluded some non-Swedish residents working for Swedish companies. Moreover, the HPA database is likely biased towards healthier individuals in the working population, as those on sick leave have not been offered participation. The last two years coincided with the COVID-19 pandemic, which may have affected the ability to offer and participate in an HPA. Other limitations include the lack of information on the specific steps leading up to an employee being offered participation in an HPA. In Sweden, general health assessments are one of the most sought-after services from occupational health services, yet they are not legally mandated and operated on the free market. This results in variation among organizations in terms of offering an HPA to their employees. Unfortunately, we have no information on this aspect, which limits our ability to calculate participation rates and draw conclusions about those who were offered but declined participation. Albeit the standardized education of the HPA coaches, we lack detailed information on the delivery of the dialogue.

### Concluding remarks

Although participants attending the HPA throughout the years have not fully been representative of the Swedish workforce, the HPA has reached working professionals across various sociodemographics and work organizations, including previously defined hard-to-reach groups, with small magnitude in under- or overrepresentation for most of the subgroups studied. This may be compared with samples of well-known and often-cited observational studies which most often are not representative of the general population and with a clear 'healthy worker' effect (45). The present findings may provide important input for future targeted interventions in the Swedish working population to better reach and serve underrepresented and high-risk groups. They may also serve as a comparative example for employers and health services in other countries when making their own decisions on groups to target. Preventive initiatives without targeted efforts may primarily attract already healthy individuals, thus increasing the risk of health inequalities (46). For instance, we saw that those with low education, low income, and in low-skilled occupations were underrepresented. These groups have been shown to be at higher risk of unhealthy lifestyle behaviors (17), cardiovascular disease (30), and increased sickness absence (47). Similarly, young and middle-aged women, previously identified as vulnerable to burnout (48), were also underrepresented. Future research directions include a deeper investigation into the potential short- and long-term effects of participating in health assessments within the occupational context, as well as exploring the perspectives of employees, employers, and occupational health services on the barriers and facilitators to participation and the delivery of health assessments.

## Supplementary material

Supplementary material
