# Supplementary material for: In or out of reach? Long-term trends in the reach of health assessments in the Swedish occupational setting
Source: Scand J Work Environ Health. 2024 Nov 28;50(8):641–52. doi: 10.5271/sjweh.4192 (PMC11626603; doi:10.5271/sjweh.4192)
Supplement: Supplementary material [file SJWEH-50-641-S001.pdf]

In or out of reach? Long-term trends in the reach of health assessments in the Swedish occupational setting[1]

by Elin Ekblom-Bak, PhD,[2] Magnus Lindwall, PhD, Linnea Eriksson, MSc, Andreas Stenling, PhD, Magnus Svartengren, MD, PhD, Robert Lundmark, PhD, Lena Kallings, PhD, Erik Hemmingsson, PhD, Daniel Väisänen, PhD

1. Supplementary material
2. Corresponding author: Elin Ekblom-Bak, Department of Physical Activity and Health, The Swedish School of Sport and Health Sciences, Box 5626, 114 86 Stockholm, Sweden [E-mail: Elin.EkblomBak@gih.se]

<u>List of content</u>

Supplementary Figure 1. Flowchart of the inclusion for the study sample and characteristics of included vs excluded individuals.

Supplementary Table 1. Categorization of self-reported variables from the HPA questionnaire.

Supplementary Table 2. Classification of major and sub-major occupational groups by classification versions.

Supplementary Table 3. Anthropometric and lifestyle characteristics of the Health Profile Assessment population (n=418 286), undertaking a health test as part of occupational health services in Sweden between 1995 to 2021.

Supplementary Table 4. Sociodemographic characteristics for the comparative population.

Supplementary Table 5. Relative difference in sociodemographic characteristics, HPA vs Comparative population.

Supplementary Table 6. Work organization characteristics for the comparative population.

Supplementary Table 7. Relative difference in work organization characteristics, HPA vs Comparative population.

Supplementary Table 8. Description of all combined groups in underrepresented, represented, and overrepresented clusters in private sector.

Supplementary Table 9. Description of all combined groups in underrepresented, represented, and overrepresented clusters in public sector.

Supplementary Figure 2. Characteristics of individuals in the underrepresented and overrepresented groups in the HPA population.

Supplementary Table 10. Percentages in sex and age groups by occupations in underrepresented, represented, and overrepresented clusters, stratified by ownership sector.

Supplementary Table 11. Sociodemographic and work organization characteristics by clusters of underrepresented, represented, and overrepresented groups in the private sector.

Supplementary Table 12. Sociodemographic and work organization characteristics by clusters of underrepresented, represented, and overrepresented groups in the public sector.

Supplementary Table 13. Detailed occupational codes on the unit level and their descriptions in groups of underrepresented, represented, and overrepresented clusters by ownership sector.

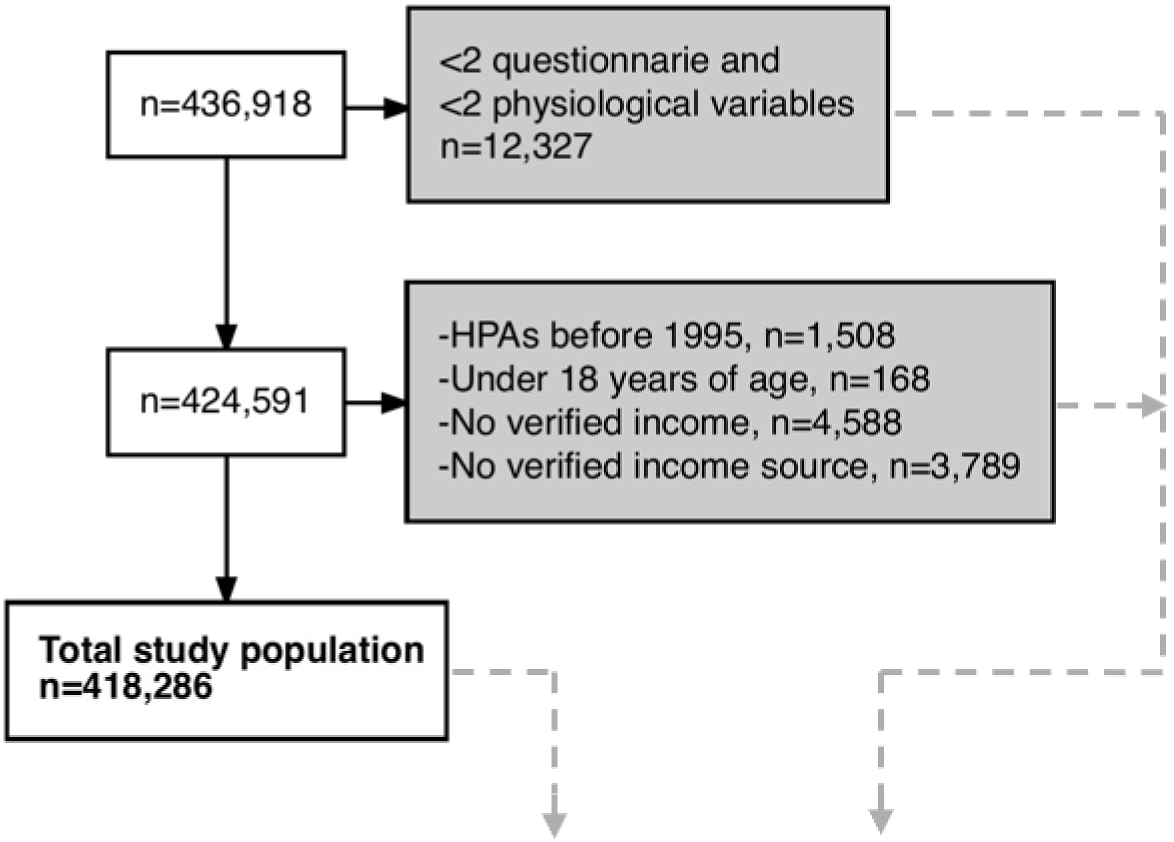

	Included (n=418,286)	Excluded (n=18,632)
% Women	45%	44%
% 18-35 years	31%	30%
% 36-50 years	41%	38%
% 51-65 years	29%	22%
% >65 years	0.3%	9%
% Tertiary education	43%	22%

Supplementary Figure 1. Flowchart of the inclusion for the study sample and characteristics of included vs excluded individuals.

Supplementary Table 1. Categorization of self-reported variables from the HPA questionnaire.

Variable and statement	Original categories	Aggregated categories
Exercise habits<br>*I exercise for the purpose of maintaining/improving my physical fitness, health and well-being...*	○ Never<br>○ Sometimes<br>○ 1-2 times/week<br>○ 3-5 times/week<br>○ At least 6 times/week	○ Never/Occasionally<br>○ 1-2 times/week<br>○ ≥ 3 times/week
Physical work situation	○ Sedentary work with elements of activity<br>○ Physically active work<br>○ Occasionally physically demanding work<br>○ Occasionally highly physically demanding work	○ Sedentary work<br>○ Physically active work<br>○ Physically demanding work
Smoking habits<br>*I smoke...*	○ At least 20 cig/day<br>○ 11-19 cig/day<br>○ 1-10 cig/day<br>○ Occasionally<br>○ Never	○ Daily<br>○ Occasionally<br>○ Never
Perceived health<br>*I perceive my physical and mental health as...*	○ Very poor<br>○ Poor<br>○ Neither good nor bad<br>○ Good<br>○ Very good	○ Poor<br>○ Neutral<br>○ Good

Supplementary Table 2. Classification of major and sub-major occupational groups by classification versions.

**Version**		**Occupation**
**SSYK2012***	**SSYK96***	**Occupation**
0	0	Military
1	1	Managers
21	21	Science and engineering
22	22	Health care
23	23	Education
24-26	24	Other professionals
3	3	Associate professionals
4	4	Administration and customer service
53	513	Personal care
51-52	51-52 (excl. 513)	Service and shop sales
6	6	Agriculture and forestry
71	71	Building
72-76	72-76	Manufacturing
83	83	Transport
80-82, 84-89	80-82, 84-89	Mechanical manufacturing
91	912	Cleaners
92-96	91-93 (excl. 912)	Other elementary occupations

*SSYK2012= Swedish Standard Classification of Occupations from 2012

*SSYK96=Swedish Standard Classification of Occupations from 1996

Supplementary Table 3. Anthropometric and lifestyle characteristics of the Health Profile Assessment population (n=418 286), undertaking a health test as part of occupational health services in Sweden between 1995 to 2021.

**Variables**	**1995-1999 (*n*=13,272)**	**2000-2004 (*n*=63,446)**	**2005-2009 (*n*=122,295)**	**2010-2014 (*n*=114,468)**	**2015-2019 (*n*=85,950)**	**2020-2021 (*n*=18,855)**
**Sex**						
Men	7,005 (53%)	30,435 (48%)	63,576 (52%)	64,428 (56%)	51,387 (60%)	12,134 (64%)
Women	6,267 (47%)	33,011 (52%)	58,719 (48%)	50,040 (44%)	34,563 (40%)	6,721 (36%)
Missing	0 (0.0%)	0 (0.0%)	0 (0.0%)	0 (0.0%)	0 (0.0%)	0 (0.0%)
**Age (year)**	41.9 (10.3)	43.2 (11.1)	43.6 (11.3)	42.6 (11.6)	42.1 (11.7)	41.2 (11.9)
Missing	0 (0.0%)	0 (0.0%)	0 (0.0%)	0 (0.0%)	0 (0.0%)	0 (0.0%)
**BMI (kg/m^2)**	25.2 (3.7)	25.4 (4.0)	25.7 (4.2)	25.9 (4.3)	26.2 (4.6)	26.4 (4.8)
Men	25.7 (3.5)	26.0 (3.6)	26.3 (3.8)	26.5 (4.0)	26.7 (4.3)	27.0 (4.6)
Women	24.5 (3.9)	24.9 (4.3)	25.1 (4.5)	25.2 (4.7)	25.4 (4.9)	25.5 (5.0)
Missing	6 (0.0%)	107 (0.2%)	274 (0.2%)	353 (0.3%)	376 (0.4%)	119 (0.6%)
**BMI categories (kg/m^2)**						
Underweight and normal weight <25	7,181 (54%)	32,732 (52%)	59,448 (49%)	53,529 (47%)	38,521 (45%)	8,142 (43%)
Overweight ≥25 & <30	4,785 (36%)	23,207 (37%)	45,814 (37%)	43,278 (38%)	32,283 (38%)	7,047 (37%)
Obesity ≥30	1,300 (9.8%)	7,400 (12%)	16,759 (14%)	17,308 (15%)	14,770 (17%)	3,547 (19%)
Missing	6 (0.0%)	107 (0.2%)	274 (0.2%)	353 (0.3%)	376 (0.4%)	119 (0.6%)
**Estimated VO_2max (ml/min/kg)**	38.0 (10.0)	36.8 (9.8)	36.4 (9.9)	36.7 (10.2)	36.0 (10.1)	35.9 (10.0)
Men	38.6 (10.0)	37.6 (9.9)	36.8 (9.8)	36.7 (10.0)	36.0 (10.0)	35.7 (9.8)
Women	37.3 (9.9)	36.1 (9.7)	35.9 (9.9)	36.6 (10.4)	36.0 (10.2)	36.2 (10.2)
Missing	2,321 (17%)	11,592 (18%)	24,197 (20%)	20,264 (18%)	18,228 (21%)	4,572 (24%)
**Estimated VO_2max categories (ml/min/kg)**						
Moderate/High ≥32	7,666 (58%)	33,935 (53%)	62,412 (51%)	60,295 (53%)	41,746 (49%)	8,762 (46%)
Low <32	3,285 (25%)	17,919 (28%)	35,686 (29%)	33,909 (30%)	25,976 (30%)	5,521 (29%)
Missing	2,321 (17%)	11,592 (18%)	24,197 (20%)	20,264 (18%)	18,228 (21%)	4,572 (24%)
**Exercise habits**						
≥3 times/week	2,228 (17%)	17,553 (28%)	38,510 (31%)	39,623 (35%)	30,802 (36%)	7,843 (42%)
1 to 2 times/week	4,759 (36%)	23,135 (36%)	42,423 (35%)	36,568 (32%)	25,940 (30%)	5,671 (30%)
Occasionally/Never	6,284 (47%)	22,757 (36%)	41,356 (34%)	38,255 (33%)	29,048 (34%)	5,321 (28%)
Missing	1 (0.0%)	1 (0.0%)	6 (0.0%)	22 (0.0%)	160 (0.2%)	20 (0.1%)
**Physical work situation**						

Sedentary work	5,525 (42%)	32,536 (51%)	72,530 (59%)	64,064 (56%)	43,662 (51%)	10,649 (56%)
Physically active work	3,361 (25%)	15,475 (24%)	31,439 (26%)	22,313 (19%)	16,893 (20%)	4,020 (21%)
Physically demanding work	2,086 (16%)	8,479 (13%)	16,834 (14%)	16,662 (15%)	11,837 (14%)	3,849 (20%)
Missing	2,300 (17%)	6,956 (11%)	1,492 (1.2%)	11,429 (10.0%)	13,558 (16%)	337 (1.8%)
**Smoking habits**						
Never	10,543 (79%)	49,407 (78%)	99,245 (81%)	94,910 (83%)	71,599 (83%)	15,896 (84%)
Occasionally	1,356 (10%)	5,042 (7.9%)	8,699 (7.1%)	9,149 (8.0%)	7,783 (9.1%)	1,650 (8.8%)
Daily (≥1 cig/day)	1,371 (10%)	8,990 (14%)	14,336 (12%)	10,382 (9.1%)	6,519 (7.6%)	1,297 (6.9%)
Missing	2 (0.0%)	7 (0.0%)	15 (0.0%)	27 (0.0%)	49 (0.1%)	12 (0.1%)
**Perceived health**						
Very good/Good	7,215 (54%)	35,888 (57%)	70,654 (58%)	66,574 (58%)	48,376 (56%)	10,392 (55%)
Neither good nor bad	3,698 (28%)	17,077 (27%)	31,551 (26%)	27,185 (24%)	22,570 (26%)	5,278 (28%)
Very poor/Poor	771 (5.8%)	3,932 (6.2%)	6,809 (5.6%)	6,677 (5.8%)	6,108 (7.1%)	1,481 (7.9%)
Missing	1,588 (12%)	6,549 (10%)	13,281 (11%)	14,032 (12%)	8,896 (10%)	1,704 (9.0%)
**Episode of sickness absence (>14 days)**						
No	12,226 (92%)	57,200 (90%)	112,064 (92%)	106,503 (93%)	79,335 (92%)	17,511 (93%)
Yes	1,046 (7.9%)	6,246 (9.8%)	10,231 (8.4%)	7,965 (7.0%)	6,615 (7.7%)	1,344 (7.1%)
Missing	0 (0.0%)	0 (0.0%)	0 (0.0%)	0 (0.0%)	0 (0.0%)	0 (0.0%)

Values are presented as *n* (%) or mean (*SD*).

Supplementary Table 4. Sociodemographic characteristics for the comparative population.

Variables	1995-1999	2000-2004	2005-2009	2010-2014	2015-2019	2020-2021
**Sex**						
Men	2,563,369 (52%)	2,649,074 (52%)	2,786,183 (52%)	2,919,097 (52%)	3,118,819 (52%)	2,827,668 (52%)
Women	2,398,758 (48%)	2,482,377 (48%)	2,609,213 (48%)	2,739,478 (48%)	2,893,010 (48%)	2,659,159 (48%)
**Age group**						
18-35	1,978,437 (40%)	1,933,420 (38%)	2,000,152 (37%)	2,132,008 (38%)	2,271,130 (38%)	1,993,066 (36%)
36-50	1,567,477 (32%)	1,555,719 (30%)	1,591,336 (29%)	1,645,975 (29%)	1,679,415 (28%)	1,637,419 (30%)
51-65	1,254,351 (25%)	1,404,560 (27%)	1,467,911 (27%)	1,437,634 (25%)	1,481,930 (25%)	1,433,923 (26%)
>65	161,862 (3%)	237,752 (5%)	335,997 (6%)	442,958 (8%)	579,354 (10%)	422,419 (8%)
**Place of birth**						
Sweden	4,393,085 (89%)	4,477,033 (87%)	4,622,834 (86%)	4,714,301 (83%)	4,752,698 (79%)	4,271,386 (78%)
Europe	384,601 (8%)	403,465 (8%)	437,697 (8%)	485,271 (9%)	562,286 (9%)	511,087 (9%)
Outside Europe	183,952 (4%)	250,221 (5%)	334,013 (6%)	458,720 (8%)	696,240 (12%)	703,709 (13%)
**Civil status**						
Partner	2,276,349 (46%)	2,216,474 (43%)	2,246,554 (42%)	2,288,452 (40%)	2,408,361 (40%)	2,181,551 (40%)
Single	2,685,776 (54%)	2,914,977 (57%)	3,148,842 (58%)	3,370,123 (60%)	3,603,468 (60%)	3,305,276 (60%)
**Municipality**						
Metropolitan municipalities	1,470,565 (30%)	1,554,281 (30%)	1,666,140 (31%)	1,830,080 (32%)	2,011,942 (33%)	1,865,029 (34%)
Dense municipalities	2,119,204 (43%)	2,311,498 (45%)	2,421,880 (45%)	2,510,950 (44%)	2,646,629 (44%)	2,414,990 (44%)
Rural municipalities	1,173,966 (24%)	1,258,604 (25%)	1,305,889 (24%)	1,317,545 (23%)	1,353,258 (23%)	1,206,808 (22%)
**Education**						
Primary	1,271,406 (26%)	1,094,756 (21%)	1,015,947 (19%)	909,331 (16%)	846,142 (14%)	591,793 (11%)
Secondary	2,366,570 (48%)	2,464,163 (48%)	2,548,658 (47%)	2,630,767 (46%)	2,684,200 (45%)	2,428,566 (44%)

Tertiary	1,278,975 (26%)	1,533,368 (30%)	1,780,386 (33%)	2,051,391 (36%)	2,366,372 (39%)	2,376,939 (43%)
**Occupation**						
Military	··	10,206 (0.2%)	11,870 (0.2%)	12,469 (0.2%)	8,875 (0.1%)	··
Managers	··	149,917 (3%)	235,000 (4%)	208,746 (4%)	262,154 (4%)	297,336 (5%)
Science and engineering	··	100,457 (2%)	154,838 (3%)	135,097 (2%)	129,951 (2%)	148,572 (3%)
Health care	··	61,003 (1%)	89,677 (2%)	92,439 (2%)	206,751 (3%)	227,059 (4%)
Education	··	167,102 (3%)	232,269 (4%)	204,973 (4%)	332,430 (6%)	347,668 (6%)
Other professionals	··	169,303 (3%)	266,741 (5%)	247,427 (4%)	412,672 (7%)	499,922 (9%)
Associate professionals	··	514,539 (10%)	748,477 (14%)	609,363 (11%)	564,999 (9%)	605,089 (11%)
Administration and customer service	··	290,414 (6%)	402,408 (7%)	305,169 (5%)	364,404 (6%)	395,957 (7%)
Personal care	··	413,218 (8%)	602,030 (11%)	498,401 (9%)	638,582 (11%)	676,381 (12%)
Service and shop sales	··	225,488 (4%)	368,701 (7%)	335,070 (6%)	478,135 (8%)	512,589 (9%)
Agriculture and forestry	··	23,503 (0.5%)	62,984 (1%)	52,679 (0.9%)	100,903 (2%)	101,650 (2%)
Building	··	142,365 (3%)	224,625 (4%)	195,666 (3%)	175,183 (3%)	195,527 (4%)
Manufacturing	··	123,356 (2%)	164,431 (3%)	138,173 (2%)	229,727 (4%)	244,308 (4%)
Transport	··	99,265 (2%)	155,530 (3%)	137,712 (2%)	152,119 (3%)	176,583 (3%)
Mechanical manufacturing	··	199,391 (4%)	274,100 (5%)	177,382 (3%)	153,259 (3%)	163,033 (3%)
Cleaners	··	64,198 (1%)	95,631 (2%)	82,310 (1%)	105,040 (2%)	109,912 (2%)
Other elementary occupations	··	150,577 (3%)	223,182 (4%)	180,691 (3%)	198,175 (3%)	194,316 (4%)
**Income (% of median)**						
<60%	1,962,815 (40%)	2,048,099 (40%)	2,183,412 (40%)	2,269,880 (40%)	2,298,584 (38%)	1,796,406 (33%)
60 to <80%	371,699 (7%)	372,312 (7%)	382,857 (7%)	412,125 (7%)	477,346 (8%)	466,980 (9%)
80 to <120%	1,090,382 (22%)	1,139,961 (22%)	1,186,439 (22%)	1,268,238 (22%)	1,412,935 (24%)	1,456,775 (27%)
120 to <200%	1,245,851 (25%)	1,253,157 (24%)	1,306,258 (24%)	1,360,813 (24%)	1,477,728 (25%)	1,438,317 (26%)
≥200%	291,380 (6%)	317,922 (6%)	336,430 (6%)	347,519 (6%)	345,236 (6%)	328,349 (6%)
**Number of income sources**						

1	3,603,546 (73%)	3,597,017 (70%)	3,738,722 (69%)	3,925,419 (69%)	4,100,482 (68%)	3,905,498 (71%)
2 to 3	1,123,971 (23%)	1,249,675 (24%)	1,294,078 (24%)	1,351,916 (24%)	1,517,605 (25%)	1,330,181 (24%)
>3	116,172 (2%)	127,997 (2%)	135,167 (3%)	142,308 (3%)	160,817 (3%)	120,403 (2%)
**Contractual temporariness**						
<3 years	2,289,551 (53%)	2,453,252 (55%)	2,450,521 (53%)	2,615,316 (53%)	3,032,429 (56%)	2,759,794 (54%)
>=3 years	2,007,343 (47%)	2,013,455 (45%)	2,190,314 (47%)	2,299,890 (47%)	2,348,339 (44%)	2,395,344 (46%)

Values are presented as *n* (%).

·· indicates missing values.

Supplementary Table 5. Relative difference in sociodemographic characteristics, HPA vs Comparative population.

Variables	1995-1999	2000-2004	2005-2009	2010-2014	2015-2019	2020-2021
**Sex**						
Men	2.3%	-7.0%	0.8%	9.2%	15.1%	24.8%
Women	-2.5%	7.5%	-0.8%	-9.8%	-16.3%	-26.4%
**Age group**						
18-35	-26.0%	-28.2%	-30.5%	-25.1%	-16.4%	-0.8%
36-50	42.1%	33.7%	41.1%	47.1%	40.7%	21.5%
51-65	1.1%	18.0%	18.7%	11.8%	15.5%	3.1%
>65	-99.1%	-97.4%	-95.0%	-92.5%	-92.9%	-90.3%
**Place of birth**						
Sweden	7.2%	6.0%	6.3%	7.7%	10.4%	9.3%
Europe	-47.9%	-34.3%	-26.1%	-24.2%	-21.7%	-12.2%
Outside Europe	-72.0%	-51.9%	-53.0%	-53.5%	-53.3%	-47.5%
**Civil status**						
Partner	15.1%	19.0%	20.0%	17.1%	12.5%	4.1%
Single	-12.8%	-14.4%	-14.2%	-11.6%	-8.4%	-2.7%
**Municipality**						
Metropolitan municipalities	-21.7%	-21.0%	-17.2%	-11.5%	-5.6%	-0.3%
Dense municipalities	19.5%	13.0%	11.6%	9.5%	6.0%	2.9%
Rural municipalities	7.2%	2.0%	0.3%	-2.1%	-3.3%	-5.3%
**Education**						
Primary	-40.3%	-44.8%	-43.4%	-47.7%	-53.5%	-46.5%
Secondary	1.9%	0.3%	0.9%	0.5%	1.9%	7.1%
Tertiary	39.4%	33.6%	25.8%	23.0%	20.8%	5.8%
**Occupation**						
Military	..	-57.3%	-51.2%	-71.9%	-90.7%	
Managers	..	60.3%	65.3%	51.3%	71.0%	62.2%
Science and engineering	..	135.9%	122.1%	198.8%	292.6%	162.8%
Health care	..	33.5%	-3.5%	-22.1%	-43.3%	-54.0%
Education	..	37.2%	-4.7%	-35.8%	-44.3%	-68.3%
Other professionals	..	157.9%	64.0%	60.2%	57.6%	4.9%
Associate professionals	..	113.0%	70.9%	60.3%	92.4%	77.5%
Administration and customer service	..	73.6%	28.1%	16.0%	38.3%	24.4%

Personal care	··	9.4%	-13.1%	-34.5%	-49.4%	-71.4%
Service and shop sales	··	-39.5%	-56.0%	-52.8%	-49.9%	-56.3%
Agriculture and forestry	··	-12.2%	-41.1%	-32.1%	-57.5%	-47.2%
Building	··	-0.5%	8.5%	32.7%	67.9%	117.9%
Manufacturing	··	30.6%	45.8%	54.7%	83.9%	143.6%
Transport	··	-38.6%	-44.1%	-44.2%	-10.2%	-3.7%
Mechanical manufacturing	··	46.0%	80.0%	160.4%	157.7%	124.4%
Cleaners	··	34.7%	-19.3%	-47.0%	-36.5%	-64.4%
Other elementary occupations	··	-21.4%	-30.8%	-38.1%	-51.9%	-61.4%
**Income (% of median)**						
<60%	-89.3%	-85.3%	-88.1%	-84.4%	-88.7%	-86.9%
60 to <80%	-25.5%	-19.2%	-25.8%	-34.3%	-41.7%	-45.4%
80 to <120%	36.0%	41.7%	35.5%	17.7%	21.0%	17.1%
120 to <200%	96.0%	85.3%	95.0%	102.6%	104.6%	86.4%
≥200%	89.1%	86.2%	106.9%	125.7%	114.8%	85.8%
**Number of income sources**						
1	7.5%	10.2%	10.9%	10.5%	6.9%	4.6%
2 to 3	-11.1%	-13.8%	-10.3%	-8.5%	1.3%	0.1%
>3	-23.6%	-33.7%	-37.3%	-45.2%	-44.0%	-41.9%
**Contractual temporariness**						
<3 years	-27.2%	-19.4%	-26.6%	-25.8%	-12.8%	-1.7%
>=3 years	31.0%	23.6%	29.7%	29.4%	16.5%	1.9%

·· indicates missing values.

Supplementary Table 6. Work organization characteristics for the comparative population.

Variables	1995-1999	2000-2004	2005-2009	2010-2014	2015-2019	2020-2021
**Ownership sector**						
Private	2,968,998 (60%)	3,273,300 (64%)	3,568,214 (66%)	3,847,505 (68%)	4,071,092 (68%)	3,694,756 (67%)
Public regional	1,452,493 (29%)	1,335,040 (26%)	1,333,946 (25%)	1,340,376 (24%)	1,463,360 (24%)	1,345,558 (25%)
Public governmental	503,949 (10%)	460,459 (9%)	422,194 (8%)	417,248 (7%)	427,303 (7%)	403,851 (7%)
**Number of employees**						
1 to 9	898,075 (18%)	918,880 (18%)	990,811 (18%)	1,114,594 (20%)	1,141,586 (19%)	1,196,698 (22%)
10 to 49	1,044,840 (21%)	1,118,078 (22%)	1,184,184 (22%)	1,266,787 (22%)	1,374,974 (23%)	1,456,754 (27%)
50 to 249	977,117 (20%)	1,074,850 (21%)	1,111,941 (21%)	1,129,752 (20%)	1,287,873 (21%)	1,375,960 (25%)
≥250	728,878 (15%)	743,088 (14%)	734,371 (14%)	752,763 (13%)	859,059 (14%)	964,509 (18%)
**Economic sector**						
Agriculture, forestry, and fishing	116,815 (2%)	117,523 (2%)	133,476 (2%)	162,008 (3%)	161,626 (3%)	127,052 (2%)
Mining and quarrying	10,611 (0.2%)	8,404 (0.2%)	8,855 (0.2%)	9,795 (0.2%)	9,673 (0.2%)	9,614 (0.2%)
Manufacturing	787,327 (16%)	752,384 (15%)	686,298 (13%)	590,040 (10%)	551,817 (9%)	509,129 (9%)
Construction	256,665 (5%)	264,821 (5%)	317,168 (6%)	359,809 (6%)	394,175 (7%)	379,267 (7%)
Wholesale and retail trade; repair of motor vehicles and motorcycles	588,751 (12%)	613,078 (12%)	651,833 (12%)	672,249 (12%)	696,767 (12%)	635,751 (12%)
Transportation and storage	216,900 (4%)	225,170 (4%)	257,678 (5%)	274,076 (5%)	277,045 (5%)	255,041 (5%)
Accommodation and food service activities	170,400 (3%)	183,267 (4%)	218,967 (4%)	257,551 (5%)	274,812 (5%)	211,505 (4%)
Information and communication	243,353 (5%)	253,321 (5%)	209,262 (4%)	192,802 (3%)	226,966 (4%)	230,670 (4%)
Financial and insurance activities	95,953 (2%)	95,042 (2%)	94,666 (2%)	95,894 (2%)	100,518 (2%)	100,178 (2%)
Real estate activities	105,749 (2%)	107,786 (2%)	118,471 (2%)	124,682 (2%)	144,715 (2%)	125,309 (2%)

Professional, scientific, and technical activities	357,567 (7%)	438,169 (9%)	368,915 (7%)	322,218 (6%)	356,646 (6%)	336,983 (6%)
Administrative and support service activities	11,160 (0.2%)	12,542 (0.2%)	196,405 (4%)	358,151 (6%)	386,561 (6%)	346,158 (6%)
Public administration and defense; compulsory social security	256,177 (5%)	257,785 (5%)	267,130 (5%)	279,748 (5%)	313,254 (5%)	322,920 (6%)
Education	351,588 (7%)	459,034 (9%)	530,519 (10%)	574,706 (10%)	633,557 (11%)	590,790 (11%)
Human health and social work activities	893,451 (18%)	836,400 (16%)	828,690 (15%)	880,074 (16%)	962,868 (16%)	891,401 (16%)
Arts, entertainment, and recreation	146,091 (3%)	166,937 (3%)	147,244 (3%)	127,461 (2%)	136,941 (2%)	116,792 (2%)
Other service activities	106,306 (2%)	109,441 (2%)	144,335 (3%)	173,473 (3%)	177,946 (3%)	143,661 (3%)
Activities of extraterritorial organizations and bodies	418 (0.0%)	438 (0.0%)	697 (0.0%)	820 (0.0%)	989 (0.0%)	950 (0.0%)
Activities of households as employers; undifferentiated goods- and services-producing activities of households for own use	16 (0.0%)	3 (0.0%)	7 (0.0%)	14 (0.0%)	15 (0.0%)	12 (0.0%)
Electricity, gas, steam, and air supply	28,518 (0.6%)	23,316 (0.5%)	25,237 (0.5%)	25,270 (0.4%)	25,805 (0.4%)	26,148 (0.5%)
Water supply; sewerage, waste management, and remediation activities	14,318 (0.3%)	16,289 (0.3%)	18,203 (0.3%)	20,241 (0.4%)	22,427 (0.4%)	23,133 (0.4%)
**Operating profit (% of median)**						

>500%	550,374 (11%)	981,807 (19%)	1,070,928 (20%)	1,140,692 (20%)	1,177,439 (20%)	1,091,029 (20%)
0% to 500%	657,933 (13%)	1,137,498 (22%)	1,398,787 (26%)	1,535,561 (27%)	1,680,433 (28%)	1,499,427 (27%)
-500% to <0%	180,537 (4%)	335,754 (7%)	392,004 (7%)	439,461 (8%)	474,144 (8%)	409,911 (7%)
<-500%	184,221 (4%)	389,520 (8%)	277,891 (5%)	302,169 (5%)	271,941 (5%)	295,213 (5%)
**Operating profit margin**						
>5%	633,982 (13%)	1,126,516 (22%)	1,386,034 (26%)	1,517,319 (27%)	1,623,774 (27%)	1,657,048 (30%)
0% to 5%	574,329 (12%)	993,010 (19%)	1,083,745 (20%)	1,158,943 (20%)	1,234,213 (21%)	933,528 (17%)
-5% to <0%	223,827 (5%)	396,892 (8%)	359,373 (7%)	420,248 (7%)	396,253 (7%)	312,211 (6%)
<-5%	140,927 (3%)	328,161 (6%)	310,458 (6%)	321,373 (6%)	349,717 (6%)	392,793 (7%)
**Staff turnover rate**						
<10%	1,509,929 (30%)	1,464,621 (29%)	1,591,481 (29%)	1,662,722 (29%)	1,439,966 (24%)	1,631,006 (30%)
10% to <20%	1,128,411 (23%)	1,186,653 (23%)	1,282,347 (24%)	1,366,147 (24%)	1,508,297 (25%)	1,675,351 (31%)
≥20%	806,695 (16%)	997,720 (19%)	954,171 (18%)	1,024,808 (18%)	1,513,431 (25%)	1,479,296 (27%)

Values are presented as *n* (%).

Supplementary Table 7. Relative difference in work organization characteristics, HPA vs Comparative population.

Variables	1995-1999	2000-2004	2005-2009	2010-2014	2015-2019	2020-2021
**Ownership sector**						
Private	-15.7%	-23.0%	-9.0%	1.1%	8.8%	22.1%
Public regional	19.1%	40.2%	27.2%	10.0%	-4.5%	-35.5%
Public governmental	44.5%	60.2%	6.3%	-29.7%	-57.0%	-73.9%
**Number of employees**						
1 to 9	-64.6%	-61.8%	-55.7%	-54.3%	-53.6%	-52.8%
10 to 49	14.8%	39.4%	40.7%	31.5%	41.3%	42.4%
50 to 249	65.1%	63.8%	78.6%	52.7%	58.0%	35.4%
≥250	122.8%	73.2%	51.2%	99.3%	54.6%	-5.7%
**Economic sector**						
Agriculture, forestry, and fishing	11.7%	-70.6%	-71.4%	-60.5%	-61.4%	-64.1%
Mining and quarrying	-96.5%	-35.7%	-38.5%	-29.4%	8.3%	78.1%
Manufacturing	46.5%	36.3%	86.2%	169.9%	176.5%	148.4%
Construction	-68.0%	-43.8%	-29.1%	8.2%	52.7%	148.5%
Wholesale and retail trade; repair of motor vehicles and motorcycles	-37.6%	5.9%	12.9%	-22.3%	-15.9%	3.1%
Transportation and storage	-37.3%	-23.0%	-35.7%	-33.8%	-20.8%	-21.2%
Accommodation and food service activities	-54.9%	-80.6%	-89.0%	-83.6%	-87.6%	-88.0%
Information and communication	-4.1%	-7.8%	29.6%	38.7%	3.5%	-12.0%
Financial and insurance activities	133.5%	170.7%	73.7%	29.5%	17.8%	-5.1%
Real estate activities	-3.0%	56.2%	51.1%	47.4%	68.6%	82.7%
Professional, scientific, and technical activities	-23.0%	-56.5%	-8.0%	17.7%	75.5%	77.8%
Administrative and support service activities	-46.5%	-52.4%	-61.4%	-67.3%	-61.5%	-57.8%
Public administration and defense; compulsory social security	60.2%	77.1%	15.8%	29.7%	-6.4%	-56.5%

Education	13.4%	12.1%	-4.0%	-30.9%	-50.5%	-72.1%
Human health and social work activities	-8.8%	-10.9%	-21.9%	-32.9%	-43.3%	-61.9%
Arts, entertainment, and recreation	102.6%	50.8%	-40.3%	-32.3%	-28.7%	-32.6%
Other service activities	-3.2%	-45.3%	-0.5%	-14.8%	7.2%	34.3%
Electricity, gas, steam, and air supply	165.4%	299.3%	371.1%	315.8%	150.8%	105.2%
Water supply; sewerage, waste management, and remediation activities	248.9%	84.6%	206.7%	307.3%	271.8%	270.1%
**Operating profit (% of median)**						
>500%	98.8%	73.0%	86.3%	106.8%	101.4%	93.9%
0% to 500%	-32.2%	-61.6%	-49.7%	-44.3%	-29.6%	4.6%
-500% to <0%	-0.9%	-68.4%	-44.5%	-45.5%	-29.9%	-27.7%
<-500%	172.6%	6.9%	52.0%	57.7%	52.1%	8.0%
**Operating profit margin**						
>5%	26.9%	-9.6%	5.9%	24.3%	41.6%	51.6%
0% to 5%	28.1%	12.5%	13.6%	14.6%	1.8%	25.5%
-5% to <0%	112.7%	-27.4%	-5.9%	-3.8%	7.8%	-16.4%
<-5%	45.5%	-28.8%	-2.8%	-2.9%	-8.8%	-9.8%
**Staff turnover rate**						
<10%	37.0%	43.8%	54.3%	63.7%	55.1%	51.6%
10% to <20%	58.4%	60.4%	47.4%	38.6%	61.3%	22.0%
≥20%	2.3%	-15.1%	-18.4%	-30.3%	-28.7%	-45.3%

Supplementary Table 8. Description of all combined groups in underrepresented, represented, and overrepresented clusters in private sector.

Description of the combined groups	HPA (%)	Comp. Population (%)	Difference HPA vs Comp. Pop	HPA (n)	Comp. Population (n)
**Underrepresented**					
Women, 18-35, and Service and shop sales	0.66%	4.86%	-4.20%	465	67117
Men, 18-35, and Service and shop sales	0.75%	3.39%	-2.64%	533	46783
Women, 18-35, and Personal care	0.24%	1.97%	-1.73%	169	27142
Women, 36-50, and Service and shop sales	0.42%	1.86%	-1.44%	301	25693
Men, 18-35 and Other elementary occupations	0.49%	1.57%	-1.08%	344	21616
Women, 36-50, and Personal care	0.2%	1.27%	-1.07%	140	17486
Women, 51-65, and Service and shop sales	0.31%	1.37%	-1.06%	218	18983
Women, 18-35, and Other elementary occupations	0.16%	1.22%	-1.06%	113	16888
Women, 51-65, and Personal care	0.14%	1.09%	-0.95%	96	15064
**Represented**					
Men, 36-50, and Service and shop sales	0.73%	1.54%	-0.81%	516	21273
Men, 18-35, and Administration and customer service	1.56%	2.32%	-0.76%	1105	32075
Men, 51-65, and Transport	0.90%	1.66%	-0.76%	636	22927
Men, 18-35, and Personal care	0.11%	0.79%	-0.68%	81	10842
Women, 36-50, and Cleaners	0.11%	0.68%	-0.57%	75	9423
Men, 36-50, and Transport	0.86%	1.40%	-0.54%	609	19378
Women, 18-35, and Cleaners	0.07%	0.60%	-0.53%	51	8306
Men, 51-65, and Agriculture and forestry	0.23%	0.75%	-0.52%	160	10289
Women, 18-35, and Administration and customer service	1.70%	2.20%	-0.50%	1203	30347
Men, 51-65, and Service and shop sales	0.62%	1.11%	-0.49%	443	15287
Men, 18-35, and Transport	1.01%	1.43%	-0.42%	717	19688
Men, 36-50, and Other elementary occupations	0.29%	0.70%	-0.41%	204	9618
Women, 51-65, and Cleaners	0.12%	0.47%	-0.35%	83	6530
Men, 18-35, and Cleaners	0.08%	0.40%	-0.32%	59	5516
Women, 51-65, and Health care	0.11%	0.42%	-0.31%	81	5828
Men, 36-50, and Personal care	0.05%	0.35%	-0.30%	37	4902
Women, 18-35, and Education	0.17%	0.45%	-0.28%	117	6166
Women, 36-50, and Health care	0.16%	0.44%	-0.28%	113	6055
Men, 36-50, and Cleaners	0.07%	0.32%	-0.25%	49	4426
Men, 51-65, and Other elementary occupations	0.20%	0.45%	-0.25%	145	6264

Women, 36-50, and Other elementary occupations	0.08%	0.33%	-0.25%	59	4527
Men, 51-65, and Personal care	0.03%	0.26%	-0.23%	24	3594
Women, 18-35, and Health care	0.07%	0.29%	-0.22%	49	3979
Men, 36-50, and Agriculture and forestry	0.20%	0.4%	-0.20%	141	5510
Women, 51-65, and Other elementary occupations	0.08%	0.25%	-0.17%	56	3497
Men, 18-35, and Education	0.07%	0.24%	-0.17%	51	3321
Women, 36-50, and Education	0.34%	0.51%	-0.17%	242	7039
Men, 51-65, and Cleaners	0.04%	0.20%	-0.16%	29	2700
Men, 18-35 and, Other professionals	1.73%	1.88%	-0.15%	1230	25987
Women, 51-65, and Agriculture and forestry	0.08%	0.21%	-0.13%	55	2968
Men, 51-65, and Health care	0.02%	0.16%	-0.14%	16	2160
Women, 51-6,5 and Education	0.21%	0.33%	-0.12%	147	4583
Men, 18-35, and Agriculture and forestry	0.19%	0.31%	-0.12%	133	4256
Men, 36-50, and Health care	0.03%	0.15%	-0.12%	23	2017
Women, 18-35, and Agriculture and forestry	0.07%	0.18%	-0.11%	53	2544
Women, 18-35, and Managers	0.27%	0.38%	-0.11%	191	5212
Men, 36-50, and Education	0.12%	0.22%	-0.10%	87	2983
Men, 18-35, and Health care	0.02%	0.09%	-0.07%	12	1233
Women, 51-65, and Transport	0.05%	0.11%	-0.06%	32	1538
Men, 51-65, and Education	0.08%	0.14%	-0.06%	56	1894
Women, 36-50, and Agriculture and forestry	0.10%	0.15%	-0.05%	68	2092
Men, 18-35, and Managers	0.56%	0.61%	-0.05%	397	8461
Women, 18-35, and Transport	0.12%	0.18%	-0.06%	87	2419
Women, 36-50, and Transport	0.06%	0.11%	-0.05%	45	1463
Men, 18-35, and Military	0.00%	0.02%	-0.02%	0	247
Women, 18-35, and Military	0.00%	0.00%	0.00%	0	23
Men, 36-50, and Military	0.00%	0.00%	0.00%	0	13
Men, 51-65, and Military	0.00%	0.00%	0.00%	0	9
Women, 36-50, and Military	0.00%	0.00%	0.00%	0	2
Women, 51-65, and Military	0.00%	0.00%	0.00%	0	1
Women, 51-65, and Manufacturing	0.21%	0.21%	0.00%	151	2835
Women, 36-50, and Building	0.05%	0.04%	0.01%	37	607
Women, 51-65, and Building	0.03%	0.02%	0.01%	23	312
Men, 51-65, and Building	1.54%	1.52%	0.02%	1089	21051
Women, 18-35, and Manufacturing	0.32%	0.30%	0.02%	229	4184
Women, 36-50, and Manufacturing	0.26%	0.23%	0.03%	181	3150

Women, 18-35, and Building	0.16%	0.11%	0.05%	113	1458
Women, 18-35, and Other professionals	1.33%	1.25%	0.08%	940	17260
Women, 51-65, and Managers	0.79%	0.70%	0.09%	563	9691
Women, 51-65, and Mechanical manufacturing	0.49%	0.35%	0.14%	350	4887
Women, 18-35, and Mechanical manufacturing	0.60%	0.45%	0.15%	427	6201
Men, 51-65, and Administration and customer service	0.95%	0.76%	0.19%	673	10431
Men, 36-50, and Building	2.05%	1.84%	0.21%	1450	25364
Men, 51-65, and Other professionals	1.52%	1.29%	0.23%	1079	17776
Men, 36-50, and Administration and customer service	1.33%	1.08%	0.25%	940	14933
Women, 51-65, and Science and engineering	0.50%	0.22%	0.28%	353	2988
Women, 36-50, and Mechanical manufacturing	0.64%	0.35%	0.29%	456	4835
Women, 51-65, and Other professionals	1.18%	0.85%	0.33%	836	11791
Men, 36-50, and Other professionals	2.70%	2.35%	0.35%	1914	32463
Women, 51-65, and Administration and customer service	1.74%	1.38%	0.36%	1235	19019
Women, 36-50, and Managers	1.52%	1.11%	0.41%	1075	15273
Men, 51-65, and Managers	2.29%	1.83%	0.46%	1626	25256
Women, 51-65, and Associate professionals	1.92%	1.45%	0.47%	1359	20015
**Overrepresented**					
Men, 51-65, and Manufacturing	2.75%	2.10%	0.65%	1951	29007
Women, 18-35, and Associate professionals	2.58%	1.92%	0.66%	1828	26569
Women, 36-50, and Administration and customer service	2.34%	1.59%	0.75%	1662	21981
Men, 18-35, and Building	3.20%	2.42%	0.78%	2268	33409
Women, 36-50, and Other professionals	2.29%	1.48%	0.81%	1624	20412
Men, 36-50, and Manufacturing	3.04%	2.21%	0.83%	2152	30550
Men, 36-50, and Managers	3.22%	2.33%	0.89%	2283	32210
Men, 18-35, and Mechanical manufacturing	2.65%	1.63%	1.02%	1877	22481
Men, 51-65, and Mechanical manufacturing	2.13%	1.07%	1.06%	1510	14834
Men, 51-65, and Science and engineering	1.77%	0.71%	1.06%	1255	9806
Women, 36-50, and Science and engineering	1.59%	0.52%	1.07%	1130	7116
Women, 18-35, and Science and engineering	1.57%	0.46%	1.11%	1112	6292
Women, 36-50, and Associate professionals	3.23%	2.09%	1.14%	2291	28837
Men, 36-50, and Mechanical manufacturing	2.36%	1.15%	1.21%	1676	15853
Men, 18-35, and Associate professionals	4.31%	2.93%	1.38%	3056	40396
Men, 18-35, and Science and engineering	2.20%	0.80%	1.4%	1563	11096
Men, 51-65, and Associate professionals	4.30%	2.75%	1.55%	3049	38008
Men, 18-35, and Manufacturing	4.25%	2.69%	1.56%	3015	37196

Men, 36-50, and Science and engineering	3.06%	1.12%	1.94%	2166	15446
Men, 36-50, and Associate professionals	5.90%	3.59%	2.31%	4186	49544

Supplementary Table 9. Description of all combined groups in underrepresented, represented, and overrepresented clusters in public sector.

**Description of the combined groups**	**HPA (%)**	**Comp. Population (%)**	**Difference HPA vs Comp. Pop**	**HPA (n)**	**Comp. Population (n)**
**Underrepresented**					
Women, 36-50, and Education	3.18%	5.55%	-2.37%	733	37766
Women, 51-65, and Personal care	5.56%	7.57%	-2.01%	1280	51520
Women, 51-65, and Education	2.55%	4.46%	-1.91%	586	30350
Women, 18-35, and Personal care	4.82%	6.59%	-1.77%	1110	44874
Women, 18-35, and Education	1.61%	3.24%	-1.63%	371	22070
Women, 36-50, and Health care	2.03%	3.27%	-1.24%	467	22234
Men, 18-35, and Personal care	0.82%	2.01%	-1.19%	189	13663
Women, 51-65, and Health care	1.56%	2.72%	-1.16%	360	18549
Men, 36-50, and Education	0.89%	1.77%	-0.88%	204	12047
Men, 18-35, and Education	0.62%	1.4%	-0.78%	142	9533
Men, 51-65, and Education	0.80%	1.58%	-0.78%	185	10770
Women, 36-50, and Personal care	5.90%	6.65%	-0.75%	1357	45258
**Represented**					
Men, 36-50, and Personal care	0.50%	1.10%	-0.60%	116	7497
Men, 51-65, and Personal care	0.43%	0.92%	-0.49%	98	6250
Men, 36-50, and Health care	0.45%	0.91%	-0.46%	103	6202
Men, 51-65, and Health care	0.26%	0.64%	-0.38%	59	4356
Women, 18-35, and Health care	2.38%	2.72%	-0.34%	548	18510
Men, 51-65, and Other elementary occupations	0.32%	0.65%	-0.33%	73	4395
Men, 18-35, and Military	0.00%	0.28%	-0.28%	0	1920
Men, 18-35, and Administration and customer service	0.45%	0.71%	-0.26%	103	4862
Men, 51-65, and Administration and customer service	0.34%	0.54%	-0.20%	79	3697
Men, 18-35, and Other elementary occupations	0.27%	0.45%	-0.18%	63	3094
Women, 18-35, and Other elementary occupations	0.24%	0.42%	-0.18%	56	2869
Women, 51-65, and Associate professionals	1.62%	1.78%	-0.16%	373	12134
Women, 18-35, and Service and shop sales	0.58%	0.70%	-0.12%	134	4798
Men, 36-50, and Administration and customer service	0.33%	0.45%	-0.12%	77	3067

Women, 51-65, and Other elementary occupations	0.72%	0.82%	-0.10%	165	5600
Men, 36-50, and Military	0.00%	0.07%	-0.07%	0	457
Women, 18-35, and Other professionals	2.09%	2.15%	-0.06%	482	14614
Men, 51-65, and Military	0.00%	0.04%	-0.04%	0	289
Women, 18-35, and Military	0.00%	0.03%	-0.03%	0	230
Women, 36-50, and Other elementary occupations	0.54%	0.55%	-0.01%	124	3773
Women, 18-35, and Transport	0.04%	0.05%	-0.01%	10	349
Men, 18-35, and Agriculture and forestry	0.05%	0.06%	-0.01%	12	401
Women, 36-50, and Military	0.00%	0.01%	-0.01%	0	39
Women, 51-65, and Transport	0.03%	0.03%	0.00%	6	191
Women, 51-65, and Military	0.00%	0.00%	0.00%	0	13
Women, 18-35, and Agriculture and forestry	0.05%	0.05%	0.00%	11	331
Women, 36-50, and Building	0.01%	0.01%	0.00%	2	64
Women, 18-35, and Manufacturing	0.03%	0.03%	0.00%	7	203
Women, 51-65, and Building	0.01%	0.01%	0.00%	2	50
Women, 36-50, and Transport	0.03%	0.03%	0.00%	8	225
Men, 18-35, and Cleaners	0.09%	0.08%	0.01%	21	546
Women, 51-65, and Mechanical manufacturing	0.04%	0.03%	0.01%	10	217
Men, 51-65, and Agriculture and forestry	0.12%	0.11%	0.01%	28	743
Women, 18-35, and Mechanical manufacturing	0.10%	0.08%	0.02%	23	569
Men, 18-35, and Health care	0.76%	0.74%	0.02%	175	5056
Women, 51-65, and Agriculture and forestry	0.05%	0.03%	0.02%	11	199
Women, 18-35, and Building	0.03%	0.01%	0.02%	7	68
Men, 51-65, and Cleaners	0.08%	0.06%	0.02%	19	409
Women, 51-65, and Manufacturing	0.04%	0.02%	0.02%	10	104
Men, 18-35, and Managers	0.18%	0.14%	0.04%	42	967
Women, 36-50, and Agriculture and forestry	0.08%	0.03%	0.05%	19	226
Women, 36-50, and Mechanical manufacturing	0.09%	0.04%	0.05%	21	269
Women, 36-50, and Manufacturing	0.07%	0.01%	0.06%	15	89
Men, 18-35, and Transport	0.27%	0.21%	0.06%	61	1446
Women, 18-35, and Managers	0.25%	0.20%	0.05%	58	1355
Men, 36-50, and Agriculture and forestry	0.12%	0.06%	0.06%	28	405
Men, 36-50, and Other elementary occupations	0.47%	0.40%	0.07%	108	2720
Men, 36-50, and Cleaners	0.15%	0.07%	0.08%	35	480
Men, 51-65, and Manufacturing	0.28%	0.18%	0.10%	65	1248
Men, 51-65, and Transport	0.40%	0.29%	0.11%	93	2004

Women, 51-65, and Other professionals	2.48%	2.33%	0.15%	570	15851
Women, 51-65, and Science and engineering	0.29%	0.14%	0.15%	66	943
Women, 18-35, and Cleaners	0.34%	0.19%	0.15%	78	1295
Men, 18-35, and Manufacturing	0.38%	0.21%	0.17%	88	1425
Men, 18-35, and Other professionals	0.96%	0.78%	0.18%	220	5313
Men, 36-50, and Manufacturing	0.35%	0.16%	0.19%	80	1059
Women, 36-50, and Cleaners	0.52%	0.30%	0.22%	120	2039
Men, 36-50, and Transport	0.43%	0.20%	0.23%	98	1377
Men, 51-65, and Science and engineering	0.58%	0.30%	0.28%	133	2074
**Overrepresented**					
Men, 18-35, and Building	0.38%	0.09%	0.29%	87	611
Women, 51-65, and Service and shop sales	0.86%	0.57%	0.29%	199	3914
Men, 18-35, and Service and shop sales	1.07%	0.76%	0.31%	247	5174
Men, 18-35, and Mechanical manufacturing	0.55%	0.23%	0.32%	127	1533
Women, 51-65, and Cleaners	0.73%	0.39%	0.34%	169	2669
Men, 36-50, and Building	0.44%	0.09%	0.35%	102	636
Men, 18-35, and Science and engineering	0.57%	0.21%	0.36%	131	1444
Men, 51-65, and Other professionals	1.60%	1.23%	0.37%	369	8394
Women, 36-50, and Service and shop sales	0.98%	0.61%	0.37%	225	4130
Men, 51-65, and Building	0.52%	0.14%	0.38%	120	946
Men, 18-35, and Associate professionals	1.74%	1.33%	0.41%	400	9030
Women, 51-65, and Managers	1.95%	1.45%	0.50%	448	9857
Men, 36-50, and Science and engineering	0.87%	0.37%	0.50%	201	2536
Women, 18-35, and Administration and customer service	1.62%	1.11%	0.51%	374	7527
Men, 36-50, and Other professionals	2.03%	1.51%	0.52%	467	10271
Women, 18-35, and Science and engineering	0.79%	0.25%	0.54%	181	1712
Women, 18-35, and Associate professionals	2.21%	1.67%	0.54%	508	11356
Men, 51-65, and Managers	1.48%	0.91%	0.57%	340	6224
Women, 36-50, and Science and engineering	0.93%	0.36%	0.57%	214	2451
Men, 36-50, and Mechanical manufacturing	0.80%	0.18%	0.62%	184	1255
Men, 51-65, and Mechanical manufacturing	0.86%	0.21%	0.65%	199	1437
Women, 36-50, and Associate professionals	2.74%	2.06%	0.68%	631	14043
Men, 36-50, and Managers	1.62%	0.91%	0.71%	373	6176
Women, 51-65, and Administration and customer service	2.82%	2.08%	0.74%	649	14146
Men, 51-65, and Associate professionals	2.23%	1.46%	0.77%	513	9919
Women, 36-50, and Managers	2.16%	1.39%	0.77%	498	9431

Men, 36-50, and Service and shop sales	1.52%	0.73%	0.79%	349	4940
Men, 36-50, and Associate professionals	2.57%	1.64%	0.93%	591	11198
Men, 51-65, and Service and shop sales	1.78%	0.85%	0.93%	410	5811
Women, 36-50, and Administration and customer service	2.87%	1.54%	1.33%	660	10482
Women, 36-50, and Other professionals	4.58%	3.22%	1.36%	1055	21954

Underrepresented, represented, and overrepresented clusters were identified based on a combination of three variables: sex, age group, and occupational group. The figure shows the **characteristics** of these clusters, which consist of various **combined groups** of the three variables, can be seen in Table 3. Further characteristics of these clusters are in Supplementary Table 11.

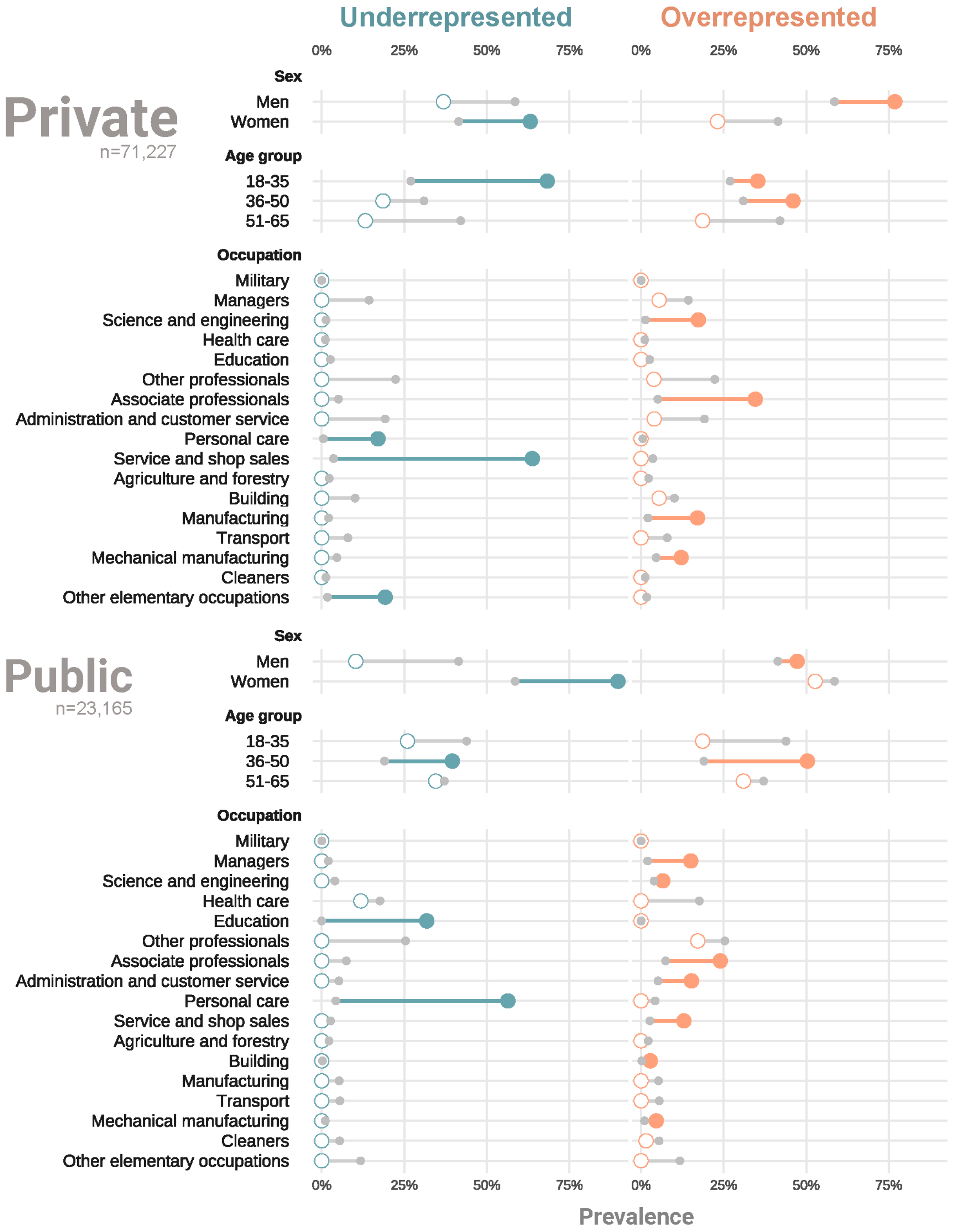

Represented cluster percentages (the cluster that has **combined groups** that is least different from the comparative population).

Underrepresented or overrepresented cluster has a higher prevalence of these subgroups in comparison to the represented cluster percentages.

Underrepresented or overrepresented cluster has a lower prevalence of these subgroups in comparison to the represented cluster percentages.

Percentage point difference between represented and other cluster percentages.

Supplementary Figure 2. Characteristics of individuals in the underrepresented (to the left) and overrepresented (to the right) groups in the HPA population presented in Supplement Tables 8 and 9, stratified on work organizations in the private sector (above) and the public sector (below). The upper panel illustrates the data for the private sector (*n*= 71,227), and the lower panel illustrates the data for the public sector (*n*=23,165). For example: In the underrepresented group(left) and the private sector (upper panel), "Service and shop sales" have a higher prevalence (solid orange dot) compared to the represented cluster (grey dot). The colored line shows the percentage point difference between the represented and underrepresented groups.

Supplementary Table 10. Percentages in sex and age groups by occupations in underrepresented, represented, and overrepresented clusters, stratified by ownership sector.

Occupation	Ownership sector	Count	Men (%)	Women (%)	Age 18-35 (%)	Age 36-50 (%)	Age 51-65 (%)
**Underrepresented**							
Other elementary occupations	Private	457	75.27%	24.73%	100%	0.00%	0.00%
Personal care	Private	405	0.00%	100%	41.73%	34.57%	23.70%
Service and shop sales	Private	1517	35.14%	64.86%	65.79%	19.84%	14.37%
Education	Public	2221	23.91%	76.09%	23.10%	42.19%	34.71%
Health care	Public	827	0.00%	100%	0.00%	56.47%	43.53%
Personal care	Public	3936	4.80%	95.20%	33.00%	34.48%	32.52%
**Represented**							
Administration and customer service	Private	5156	52.72%	47.28%	44.76%	18.23%	37.01%
Agriculture and forestry	Private	610	71.15%	28.85%	30.49%	34.26%	35.25%
Associate professionals	Private	1359	0.00%	100%	0.00%	0.00%	100%
Building	Private	2712	93.62%	6.38%	4.17%	54.83%	41.00%
Cleaners	Private	346	39.60%	60.40%	31.79%	35.84%	32.37%
Education	Private	700	27.71%	72.29%	24.00%	47.00%	29.00%
Health care	Private	294	17.35%	82.65%	20.75%	46.26%	32.99%
Managers	Private	3852	52.52%	47.48%	15.26%	27.91%	56.83%
Manufacturing	Private	561	0.00%	100%	40.82%	32.26%	26.92%
Mechanical manufacturing	Private	1233	0.00%	100%	34.63%	36.98%	28.39%
Other elementary occupations	Private	464	75.22%	24.78%	0.00%	56.68%	43.32%
Other professionals	Private	5999	70.40%	29.60%	36.17%	31.91%	31.92%
Personal care	Private	142	100%	0.00%	57.04%	26.06%	16.90%
Science and engineering	Private	353	0.00%	100%	0.00%	0.00%	100%
Service and shop sales	Private	959	100%	0.00%	0.00%	53.81%	46.19%
Transport	Private	2126	92.29%	7.71%	37.82%	30.76%	31.42%
Administration and customer service	Public	259	100%	0.00%	39.77%	29.73%	30.50%
Agriculture and forestry	Public	109	62.39%	37.61%	21.10%	43.12%	35.78%
Associate professionals	Public	373	0.00%	100%	0.00%	0.00%	100%
Building	Public	11	0.00%	100%	63.64%	18.18%	18.18%

Cleaners	Public	273	27.47%	72.53%	36.26%	56.78%	6.96%
Health care	Public	885	38.08%	61.92%	81.69%	11.64%	6.67%
Managers	Public	100	42.00%	58.00%	100%	0.00%	0.00%
Manufacturing	Public	265	87.92%	12.08%	35.85%	35.85%	28.30%
Mechanical manufacturing	Public	54	0.00%	100%	42.59%	38.89%	18.52%
Other elementary occupations	Public	589	41.43%	58.57%	20.20%	39.39%	40.41%
Other professionals	Public	1272	17.30%	82.70%	55.19%	0.00%	44.81%
Personal care	Public	214	100%	0.00%	0.00%	54.21%	45.79%
Science and engineering	Public	199	66.83%	33.17%	0.00%	0.00%	100%
Service and shop sales	Public	134	0.00%	100%	100%	0.00%	0.00%
Transport	Public	276	91.30%	8.70%	25.72%	38.41%	35.87%
**Overrepresented**							
Administration and customer service	Private	1662	0.00%	100%	0.00%	100%	0.00%
Associate professionals	Private	14410	71.42%	28.58%	33.89%	44.95%	21.16%
Building	Private	2268	100%	0.00%	100%	0.00%	0.00%
Managers	Private	2283	100%	0.00%	0.00%	100%	0.00%
Manufacturing	Private	7118	100%	0.00%	42.36%	30.23%	27.41%
Mechanical manufacturing	Private	5063	100%	0.00%	37.07%	33.1%	29.82%
Other professionals	Private	1624	0.00%	100%	0.00%	100%	0.00%
Science and engineering	Private	7226	68.97%	31.03%	37.02%	45.61%	17.37%
Administration and customer service	Public	1683	0.00%	100%	22.22%	39.22%	38.56%
Associate professionals	Public	2643	56.91%	43.09%	34.35%	46.24%	19.41%
Building	Public	309	100%	0.00%	28.16%	33.01%	38.83%
Cleaners	Public	169	0.00%	100%	0.00%	0.00%	100%
Managers	Public	1659	42.98%	57.02%	0.00%	52.50%	47.50%
Mechanical manufacturing	Public	510	100%	0.00%	24.90%	36.08%	39.02%
Other professionals	Public	1891	44.21%	55.79%	0.00%	80.49%	19.51%
Science and engineering	Public	727	45.67%	54.33%	42.92%	57.08%	0.00%
Service and shop sales	Public	1430	70.35%	29.65%	17.27%	40.14%	42.59%

Supplementary Table 11. Sociodemographic and work organization characteristics by clusters of underrepresented, represented, and overrepresented groups in the private sector.

Variable	Underrepresented	Represented	Overrepresented
**Sex**			
Men	877 (36.9%)	15,737 (58.6%)	32,001 (76.8%)
Women	1,502 (63.1%)	11,135 (41.4%)	9,647 (23.2%)
**Age group**			
18-35	1,624 (68.3%)	7,249 (27.0%)	14,715 (35.3%)
36-50	441 (18.5%)	8,323 (31.0%)	19,168 (46.0%)
51-65	314 (13.2%)	11,300 (42.1%)	7,765 (18.6%)
**Place of birth**			
Sweden	2,090 (87.9%)	23,623 (87.9%)	36,758 (88.3%)
Europe	144 (6.1%)	2,061 (7.7%)	2,813 (6.8%)
Outside of Europe	145 (6.1%)	1,188 (4.4%)	2,077 (5.0%)
**Municipality**			
Metropolitan	741 (31.1%)	9,685 (36.0%)	13,484 (32.4%)
Dense	1,055 (44.3%)	11,311 (42.1%)	18,568 (44.6%)
Rural	583 (24.5%)	5,876 (21.9%)	9,596 (23.0%)
**Education**			
Tertiary	563 (23.7%)	12,090 (45.0%)	19,277 (46.3%)
Secondary	1,618 (68.0%)	12,559 (46.7%)	20,051 (48.1%)
Primary	198 (8.3%)	2,223 (8.3%)	2,320 (5.6%)
**Occupation**			
Military	··	6 (0.02%)	··
Managers	··	3,852 (14.33%)	2,283 (5.5%)
Science and engineering	··	353 (1.31%)	7,226 (17.4%)
Health care	··	294 (1.09%)	··
Education	··	700 (2.60%)	··
Other professionals	··	5,999 (22.32%)	1,624 (3.9%)
Associate professionals	··	1,359 (5.06%)	14,404 (34.6%)
Administration and customer service	··	5,156 (19.19%)	1,662 (4.0%)
Personal care	405 (17.0%)	142 (0.53%)	··
Service and shop sales	1,517 (63.8%)	959 (3.57%)	··
Agriculture and forestry	··	610 (2.27%)	··
Building	··	2,712 (10.09%)	2,268 (5.4%)
Manufacturing	··	561 (2.09%)	7,118 (17.1%)

Transport	··	2,126 (7.91%)	··
Mechanical manufacturing	··	1,233 (4.59%)	5,063 (12.2%)
Cleaners	··	346 (1.29%)	··
Other elementary occupations	457 (19.2%)	464 (1.73%)	··
**Income (% of median)**			
≥200%	14 (0.6%)	4,492 (16.7%)	5,488 (13.2%)
120 to <200%	525 (22.1%)	13,502 (50.2%)	24,694 (59.3%)
80 to <120%	1,303 (54.8%)	7,135 (26.6%)	9,692 (23.3%)
60 to <80%	301 (12.7%)	930 (3.5%)	1,019 (2.4%)
<60%	236 (9.9%)	813 (3.0%)	755 (1.8%)
**Number of employees**			
1 to 9	464 (19.5%)	2,836 (10.6%)	3,327 (8.0%)
10 to 49	1,121 (47.1%)	9,973 (37.1%)	13,724 (33.0%)
50 to 249	699 (29.4%)	8,865 (33.0%)	13,169 (31.6%)
≥250	95 (4.0%)	5,198 (19.3%)	11,428 (27.4%)
**Economic sector**			
Agriculture, forestry, and fishing	23 (0.97%)	581 (2.16%)	242 (0.58%)
Mining and quarrying	3 (0.13%)	43 (0.16%)	133 (0.32%)
Manufacturing	236 (9.92%)	7,096 (26.41%)	17,418 (41.82%)
Construction	178 (7.48%)	3,579 (13.32%)	5,482 (13.16%)
Wholesale and retail trade; repair of motor vehicles and motorcycles	825 (34.68%)	3,415 (12.71%)	5,283 (12.68%)
Transportation and storage	63 (2.65%)	1,749 (6.51%)	1,000 (2.40%)
Accommodation and food service activities	105 (4.41%)	177 (0.66%)	80 (0.19%)
Information and communication	47 (1.98%)	2,079 (7.74%)	1,359 (3.26%)
Financial and insurance activities	10 (0.42%)	839 (3.12%)	1,027 (2.47%)
Real estate activities	85 (3.57%)	565 (2.10%)	630 (1.51%)
Professional, scientific, and technical activities	80 (3.36%)	2,473 (9.20%)	6,772 (16.26%)
Administrative and support service activities	120 (5.04%)	771 (2.87%)	655 (1.57%)
Public administration and defense; compulsory social security	2 (0.08%)	6 (0.02%)	11 (0.03%)
Education	168 (7.06%)	718 (2.67%)	113 (0.27%)
Human health and social work activities	139 (5.84%)	406 (1.51%)	160 (0.38%)
Arts, entertainment, and recreation	46 (1.93%)	323 (1.20%)	218 (0.52%)
Other service activities	229 (9.63%)	1,862 (6.93%)	910 (2.18%)
Electricity, gas, steam, and air supply	1 (0.04%)	37 (0.14%)	90 (0.22%)

Water supply; sewerage, waste management, and remediation activities	19 (0.80%)	153 (0.57%)	65 (0.16%)
**Annual staff turnover rate**			
<10%	654 (27.5%)	11,868 (44.2%)	20,939 (50.3%)
10 to <20%	893 (37.5%)	10,165 (37.8%)	15,556 (37.4%)
≥20%	832 (35.0%)	4,839 (18.0%)	5,153 (12.4%)

·· indicates missing values.

Supplementary Table 12. Sociodemographic and work organization characteristics by clusters of underrepresented, represented, and overrepresented groups in the public sector.

**Variable**	**Underrepresented**	**Represented**	**Overrepresented**
**Sex**			
Men	720 (10.3%)	2,083 (41.5%)	5,204 (47.2%)
Women	6,264 (89.7%)	2,936 (58.5%)	5,811 (52.8%)
**Age group**			
18-35	1,812 (25.9%)	2,205 (43.9%)	2,049 (18.6%)
36-50	2,761 (39.5%)	954 (19.0%)	5,550 (50.4%)
51-65	2,411 (34.5%)	1,860 (37.1%)	3,416 (31.0%)
**Place of birth**			
Sweden	5,717 (81.9%)	4,147 (82.6%)	9,834 (89.3%)
Europe	620 (8.9%)	397 (7.9%)	620 (5.6%)
Outside of Europe	647 (9.3%)	475 (9.5%)	561 (5.1%)
**Municipality**			
Metropolitan	1,025 (14.7%)	1,497 (29.8%)	3,586 (32.6%)
Dense	4,585 (65.7%)	2,672 (53.2%)	5,630 (51.1%)
Rural	1,374 (19.7%)	850 (16.9%)	1,799 (16.3%)
**Education**			
Tertiary	3,876 (55.5%)	2,852 (56.8%)	6,553 (59.5%)
Secondary	2,896 (41.5%)	1,849 (36.8%)	3,977 (36.1%)
Primary	212 (3.0%)	318 (6.3%)	485 (4.4%)
**Occupation**			
Military	..	6 (0.1%)	..
Managers	..	100 (2.0%)	1,659 (15.1%)
Science and engineering	..	199 (4.0%)	727 (6.6%)
Health care	827 (11.8%)	885 (17.6%)	..
Education	2,221 (31.8%)	..	..
Other professionals	..	1,272 (25.3%)	1,891 (17.2%)
Associate professionals	..	373 (7.4%)	2,637 (23.9%)
Administration and customer service	..	259 (5.2%)	1,683 (15.3%)
Personal care	3,936 (56.4%)	214 (4.3%)	..
Service and shop sales	..	134 (2.7%)	1,430 (13.0%)
Agriculture and forestry	..	109 (2.2%)	..
Building	..	11 (0.2%)	309 (2.8%)
Manufacturing	..	265 (5.3%)	..

Transport	··	276 (5.5%)	··
Mechanical manufacturing	··	54 (1.1%)	510 (4.6%)
Cleaners	··	273 (5.4%)	169 (1.5%)
Other elementary occupations	··	589 (11.7%)	··
**Income (% of median)**			
≥200%	139 (2.0%)	230 (4.6%)	1,254 (11.4%)
120 to <200%	1,976 (28.3%)	2,183 (43.5%)	5,525 (50.2%)
80 to <120%	3,458 (49.5%)	1,967 (39.2%)	3,468 (31.5%)
60 to <80%	870 (12.5%)	354 (7.1%)	431 (3.9%)
<60%	541 (7.7%)	285 (5.7%)	337 (3.1%)
**Number of employees**			
1 to 9	248 (3.6%)	208 (4.1%)	418 (3.8%)
10 to 49	2,425 (34.7%)	1,321 (26.3%)	2,855 (25.9%)
50 to 249	3,100 (44.4%)	2,235 (44.5%)	5,399 (49.0%)
≥250	1,211 (17.3%)	1,255 (25.0%)	2,343 (21.3%)
**Economic sector**			
Agriculture, forestry, and fishing	··	22 (0.44%)	48 (0.44%)
Mining and quarrying	··	10 (0.20%)	10 (0.09%)
Manufacturing	··	14 (0.28%)	44 (0.40%)
Construction	3 (0.04%)	174 (3.47%)	453 (4.11%)
Wholesale and retail trade; repair of motor vehicles and motorcycles	··	··	3 (0.03%)
Transportation and storage	11 (0.16%)	269 (5.36%)	302 (2.74%)
Accommodation and food service activities	··	59 (1.18%)	68 (0.62%)
Information and communication	12 (0.17%)	9 (0.18%)	34 (0.31%)
Financial and insurance activities	1 (0.01%)	3 (0.06%)	4 (0.04%)
Real estate activities	10 (0.14%)	440 (8.77%)	2,165 (19.66%)
Professional, scientific, and technical activities	10 (0.14%)	137 (2.73%)	385 (3.50%)
Administrative and support service activities	3 (0.04%)	128 (2.55%)	142 (1.29%)
Public administration and defense; compulsory social security	129 (1.85%)	1,132 (22.55%)	3,154 (28.63%)
Education	2,670 (38.23%)	372 (7.41%)	632 (5.74%)
Human health and social work activities	4,107 (58.81%)	1,482 (29.53%)	1,738 (15.78%)
Arts, entertainment, and recreation	14 (0.20%)	256 (5.10%)	630 (5.72%)
Other service activities	1 (0.01%)	12 (0.24%)	26 (0.24%)
Electricity, gas, steam, and air supply	6 (0.09%)	221 (4.40%)	495 (4.49%)

Water supply; sewerage, waste management, and remediation activities	7 (0.10%)	279 (5.56%)	682 (6.19%)
**Annual staff turnover rate**			
<10%	832 (11.9%)	1,069 (21.3%)	2,414 (21.9%)
10 to <20%	3,938 (56.4%)	2,724 (54.3%)	6,189 (56.2%)
≥20%	2,214 (31.7%)	1,226 (24.4%)	2,412 (21.9%)

·· indicates missing values.

Supplementary Table 13. Detailed occupational codes on the unit level and their descriptions in groups of underrepresented, represented, and overrepresented clusters by ownership sector.

Occupation	Private			Public		
	Underrepresented (n)	Represented (n)	Overrepresented (n)	Underrepresented (n)	Represented (n)	Overrepresented (n)
111, Legislators and senior officials	0	21	7	0	2	43
112, Managing directors and chief executives	0	312	200	0	2	53
121, Finance managers	0	414	110	0	2	95
122, Human resource managers	0	227	41	0	1	43
123, Administration and planning managers	0	82	56	0	5	85
124, Information, communication, and public relations managers	0	77	27	0	2	40
125, Sales and marketing managers	0	545	407	0	1	51
129, Administration and service managers not elsewhere classified	0	288	128	0	8	100
131, Information and communications technology service managers	0	146	137	0	2	51
132, Supply, logistics, and transport managers	0	218	181	0	0	10
133, Research and development managers	0	126	104	0	1	6
134, Architectural and engineering managers	0	301	180	0	12	175
135, Real estate and head of administration manager	0	68	36	0	5	105
136, Production managers in construction and mining	0	281	216	0	4	54
137, Production managers in manufacturing	0	330	292	0	2	29

138, Forestry and agricultural production managers	0	40	18	0	0	1
141, Primary and secondary schools and adult education managers	0	36	10	0	2	110
142, Preschool managers	0	26	0	0	0	28
149, Education managers not elsewhere classified	0	17	0	0	0	1
151, Health care managers	0	11	6	0	10	164
152, Managers in social and curative care	0	15	1	0	3	31
153, Elderly care managers	0	5	0	0	26	132
154, Managers and leaders within religious bodies	0	44	10	0	0	0
159, Other social services managers	0	19	10	0	12	286
161, Financial and insurance managers	0	75	28	0	0	1
171, Hotel and conference managers	0	13	2	0	0	1
172, Restaurant managers	0	27	11	0	2	3
173, Retail and wholesale trade managers	0	84	53	0	1	2
174, Sports, leisure, and wellness managers	0	26	6	0	0	0
179, Other services managers not elsewhere classified	0	87	59	0	0	3
211, Physicists and chemists	0	21	272	0	22	42
212, Mathematicians, actuaries, and statisticians	0	2	27	0	2	22
213, Biologists, pharmacologists, and specialists in agriculture and forestry	0	18	202	0	8	16
214, Engineering professionals	0	258	5838	0	97	238
216, Architects and surveyors	0	32	589	0	49	189
217, Designers	0	14	316	0	2	7

218, Specialists within environmental and health protection	0	23	129	0	24	228
221, Medical doctors	0	22	0	60	64	0
222, Nursing professionals	0	112	0	386	202	0
223, Nursing professionals (cont.)	0	96	0	159	40	0
224, Psychologists and psychotherapists	0	17	0	17	25	0
225, Veterinarians	0	8	0			
226, Dentists	0	13	0	87	463	0
227, Naprapaths, physiotherapists, occupational therapists	0	33	0	140	107	0
228, Specialists in health care not elsewhere classified	0	14	0	8	10	0
231, University and higher education teachers	0	29	0	238	0	0
232, Vocational education teachers	0	66	0	72	0	0
233, Secondary education teachers	0	157	0	218	0	0
234, Primary- and pre-school teachers	0	389	0	1469	0	0
235, Teaching professionals not elsewhere classified	0	76	0	365	0	0
241, Accountants, financial analysts, and fund managers	0	860	313	0	138	221
242, Organization analysts, policy administrators, and human resource specialists	0	1094	397	0	531	796
243, Marketing and public relations professionals	0	599	319	0	116	176
251, ICT architects, systems analysts, and test managers	0	2682	370	0	79	226
261, Legal professionals	0	334	75	0	116	143

262, Museum curators and librarians, and related professionals	0	22	7	0	106	114
264, Authors, journalists, and linguists	0	115	53	0	5	6
265, Creative and performing artists	0	140	47	0	33	91
266, Social work and counseling professionals	0	10	11	0	192	165
267, Religious professionals and deacons	0	271	73	0	0	0
311, Physical and engineering science technicians	0	199	4993	0	53	880
312, Construction and manufacturing supervisors	0	28	942	0	2	52
315, Ship and aircraft controllers and technicians	0	0	14	0	0	9
321, Medical and pharmaceutical technicians	0	54	536	0	25	124
323, Complementary medicine therapists and associate professionals	0	1	0	0	0	0
324, Veterinary assistants	0	0	2			
325, Dental hygienists	0	2	6	0	18	229
331, Financial and accounting associate professionals	0	464	1336	0	26	93
332, Insurance advisors, sales and purchasing agents	0	350	4149	0	23	167
333, Business services agents	0	64	712	0	63	287
334, Administrative and specialized secretaries	0	70	215	0	42	103
335, Tax and related government associate professionals	0	40	446	0	49	168
336, Police officers	0	0	1	0	4	72
341, Social work and religious associate professionals	0	16	85	0	31	132

342, Athletes, fitness instructors, and recreational workers	0	57	275	0	23	69
343, Photographers, interior decorators and entertainers	0	12	66	0	10	22
344, Driving instructors and other instructors	0	16	44	0	0	1
345, Culinary associate professionals	0	25	27	0	2	2
351, ICT operations and user support technicians	0	36	959	0	19	224
352, Broadcasting and audio-visual technicians	0	0	8	0	1	58
411, Office assistants, and other secretaries	0	2279	1233	0	128	1347
421, Croupiers, debt collectors, and related workers	0	18	3	0	0	1
422, Client information clerks	0	744	262	0	58	331
432, Stores and transport clerks	0	2153	199	0	67	31
441, Library and filing clerks	0	5	4	0	4	15
442, Postmen and postal facility workers	0	27	0	0	3	7
443, Elected representatives	0	32	9			
511, Cabin crew, guides, and related workers	11	1	0	0	0	0
512, Cooks and cold-buffet managers	83	30	0	0	48	348
513, Waiters and bartenders	55	10	0	0	3	3
514, Hairdressers, beauty and body therapists	46	2	0	0	1	9
515, Building caretakers and related workers	219	362	0	0	46	965
516, Other service-related workers	23	20	0	0	0	4
522, Shop staff	939	484	0	0	17	19
523, Cashiers and related clerks	64	2	0	0	3	7
524, Event seller and telemarketers	60	20	0	0	1	5

531, Childcare workers and teacher aides	202	35	0	678	31	0
532, Personal care workers in health services	70	11	0	1838	78	0
533, Health care assistants	37	28	0	479	44	0
534, Attendants, personal assistants, and related workers	90	69	0	478	87	0
535, Dental nurses	14	1	0	701	1	0
541, Other surveillance and security workers	52	54	0	0	19	140
611, Market gardeners and crop growers	0	353	0	0	87	0
612, Animal breeders and keepers	0	56	0	0	11	0
613, Mixed crop and animal breeders	0	33	0	0	6	0
621, Forestry and related workers	0	174	0	0	5	0
622, Aquaculture and fishery workers	0	2	0	0	0	0
711, Carpenters, bricklayers, and construction workers	0	1556	1357	0	5	251
712, Roofers, floor layers, plumbers and pipefitters	0	718	676	0	1	42
713, Painters, Lacquerers, Chimney sweepers and related trades workers	0	533	314	0	7	23
721, Sheet and structural metal workers, molders and welders, and related workers	0	28	947	0	5	0
722, Blacksmiths, toolmakers, and related trades workers	0	352	1839	0	14	0
723, Machinery mechanics and fitters	0	79	1956	0	92	0
731, Precision-instrument makers and handicraft workers	0	9	83	0	12	0
732, Printing trades workers	0	35	108	0	3	0
741, Electrical equipment installers and repairers	0	54	1981	0	110	0

742, Electronics and telecommunications installers and repairers	0	6	173	0	7	0
752, Wood treaters, cabinet-makers, and related trades workers	0	23	269	0	8	0
753, Tailors, upholsterers, and leather craftsmen	0	21	8	0	15	0
761, Butchers, bakers, and food processors	0	21	32	0	0	0
811, Mining and mineral processing plant operators	0	10	151	0	1	5
812, Metal processing and finishing plant operators	0	64	333	0	0	5
813, Machine operators, chemical and pharmaceutical products	0	61	130	0	0	0
814, Machine operators, rubber, plastic, and paper products	0	80	363	0	0	0
815, Machine operators, textile, fur, and leather products	0	44	25	0	1	1
816, Machine operators, food and related products	0	165	369	0	1	0
817, Wood processing and papermaking plant operators	0	220	1420	0	4	26
818, Other stationary plant and machine operators	0	95	386	0	4	12
819, Process control technicians	0	151	672	0	41	452
821, Assemblers	0	454	1416	0	2	12
831, Train operators and related workers	0	7	0	0	1	0
832, Car, van, and motorcycle drivers	0	55	0	0	67	0
833, Heavy truck and bus drivers	0	1027	0	0	164	0
834, Mobile plant operators	0	1094	0	0	48	0
835, Ships' deck crews and related workers	0	0	0	0	6	0
911, Cleaners and helpers	0	295	0	0	276	175

912, Washers, window cleaners, and other cleaning workers	0	56	0	0	12	2
921, Berry pickers and planters	22	20	0	0	2	0
931, Construction laborers	99	97	0	0	2	0
932, Manufacturing laborers	130	149	0	0	1	0
933, Dockers and ground personnel	67	74	0	0	57	0
941, Fast-food workers, food preparation assistants	86	60	0	0	321	0
952, Street and market vendors	0	0	0	0	0	0
961, Recycling collectors	21	15	0	0	146	0
962, Newspaper distributors, janitors, and other service workers	44	63	0	0	85	0
